# Use of *N*‐methyliminodiacetic acid boronate esters in suzuki‐miyaura cross‐coupling polymerizations of triarylamine and fluorene monomers

**DOI:** 10.1002/pola.28682

**Published:** 2017-07-03

**Authors:** Andrew B. Foster, Viktor Bagutski, Josue I. Ayuso‐Carrillo, Martin J. Humphries, Michael J. Ingleson, Michael L. Turner

**Affiliations:** ^1^ School of Chemistry University of Manchester Oxford Road Manchester M13 9PL United Kingdom; ^2^ Cambridge Display Technology Ltd Unit 12 Cardinal Park, Cardinal Way, Godmanchester Cambridgeshire PE29 2XG United Kingdom

**Keywords:** conjugated polymers, *N*‐methyliminodiacetic acid (MIDA) boronate esters, polytriarylamines, step-growth polymerization, suzuki cross‐coupling polymerization, suzuki polycondensation

## Abstract

Polytriarylamine copolymers can be prepared by Suzuki‐Miyaura cross‐coupling reactions of bis *N*‐methyliminodiacetic acid (MIDA) boronate ester substituted arylamines with dibromo arenes. The roles of solvent composition, temperature, reaction time, and co‐monomer structure were examined and (co)polymers prepared containing 9, 9‐dioctylfluorene (F8), 4‐sec‐butyl or 4‐octylphenyl diphenyl amine (TFB), and *N*, *N*′‐bis(4‐octylphenyl)‐*N*, *N*′‐diphenyl phenylenediamine (PTB) units, using a Pd(OAc)_2_/2‐dicyclohexylphosphino‐2′,6′‐dimethoxybiphenyl (SPhos) catalyst system. The performance of a di‐functionalized MIDA boronate ester monomer was compared with that of an equivalent pinacol boronate ester. Higher molar mass polymers were produced from reactions starting with a difunctionalized pinacol boronate ester monomer than the equivalent difunctionalized MIDA boronate ester monomer in biphase solvent mixtures (toluene/dioxane/water). Matrix‐assisted laser desorption/ionization mass spectroscopic analysis revealed that polymeric structures rich in residues associated with the starting MIDA monomer were present, suggesting that homo‐coupling of the boronate ester must be occurring to the detriment of cross‐coupling in the step‐growth polymerization. However, when comparable reactions of the two boronate monomers with a dibromo fluorene monomer were completed in a single phase solvent mixture (dioxane + water), high molar mass polymers with relatively narrow distribution ranges were obtained after only 4 h of reaction. © 2017 The Authors. Journal of Polymer Science Part A: Polymer Chemistry Published by Wiley Periodicals, Inc. J. Polym. Sci., Part A: Polym. Chem. **2017**, *55*, 2798–2806

## INTRODUCTION

Boronic acids and esters are widely used as intermediates in the syntheses of pharmaceuticals, natural products, and organic materials via Suzuki‐Miyaura reactions.^1^ In the materials field, these types of reactions often provide the best approach to obtain a range of conjugated polymers for electronic applications.^2^ However, the fidelity of these cross‐coupling polymerizations is often compromised by the protodeboronation of monomers or growing chains under the reaction conditions. A variety of boron protecting groups,^3^ such as trifluoroborate salts,^4^ trialkyoxyborate salts,^5^ and *N*‐methyliminodiacetic acid (MIDA) boronate esters^6^, ^7^, ^8^, ^9^, ^10^, ^11^, ^12^, ^13^, ^14^ have been used to extend the range of molecules amenable to high fidelity Suzuki‐Miyaura cross coupling. The potential for the synthesis of conjugated polymers is supported by recent work on the use of trifluoroborates for coupling reactions of electron deficient monomers.^15^

**Scheme 1:**
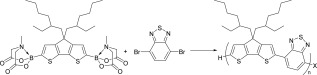
Example of Suzuki‐Miyaura copolymerization of thienyl bis MIDA boronate ester with dibromoarene.

MIDA boronate esters are of particular interest as they are cheap to synthesize, air stable, and the hydrolysis can be controlled to slowly release boronic acids for effective cross‐coupling reactions.^6^ We have reported the first use of a MIDA boronate ester protecting group on a bifunctional thienyl (AB type) monomer in Suzuki‐Miyaura polymerizations to synthesize highly regioregular poly(3‐hexylthiophene‐2,5‐diyl) (rr‐P3HT).^16^ This work was facilitated by facile production of the monomer in high yield by amine‐mediated electrophilic borylation which provides a direct route from aryl‐H to aryl‐B(OR)_2_ compounds.^17^, ^18^, ^19^, ^20^ This process produced MIDA boronate esters without requiring the synthesis and isolation of arylboronic acid intermediates, some of which can be particularly susceptible to protodeboronation.^6^, ^7^, ^21^, ^22^ The slow hydrolysis of thienyl bis MIDA boronate ester (AA type) monomers has most recently been used in Suzuki‐Miyaura copolymerizations to produce a range of thiophene containing polymers (Scheme 1).^23^

Polytriarylamines (PTAAs) are amorphous semiconducting polymers of interest in organic electronics,^24^ as they can be readily processed from solution and show stable performance in air, with moderate charge‐carrier mobilities in organic field effect transistors (up to 0.05 cm^2^ V^−1^ s^−1^). They have been used very successfully in blends with small molecule organic semiconductors to deliver very high performance, robust, reproducible organic field‐effect transistor (OFET) devices.^25^ In general, the highest performing PTAAs have bridged phenyl units, such as fluorenes or indenofluorenes in the polymer backbone and these polymers are routinely synthesized by Suzuki‐Miyaura reactions.

**Scheme 2:**
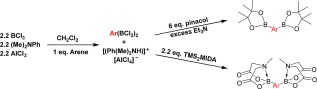
Electrophilic diborylation of arylamine monomers with protection steps. [Color figure can be viewed at wileyonlinelibrary.com]

This contribution discusses the utility of using bis MIDA boronate ester substituted arylamines to produce high molar mass arylamine (co)polymers in Suzuki‐Miyaura cross‐coupling reactions. The two step, one pot electrophilic borylation process can be used to produce both bis pinacol and bis MIDA boronate ester protected arylamine monomers at room temperature, as outlined in Scheme 2.

**Scheme 3:**
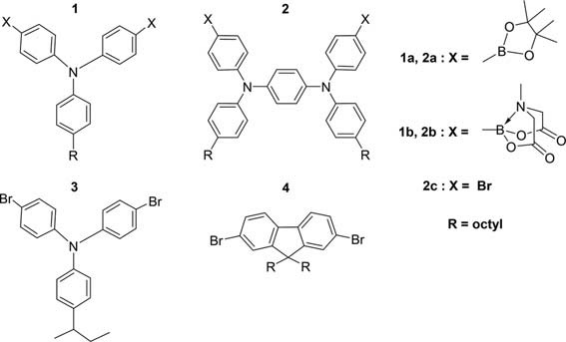
Structures of boronate and dibromo arene monomers used in the Suzuki‐Miyaura cross‐coupling reactions.

**Scheme 4:**
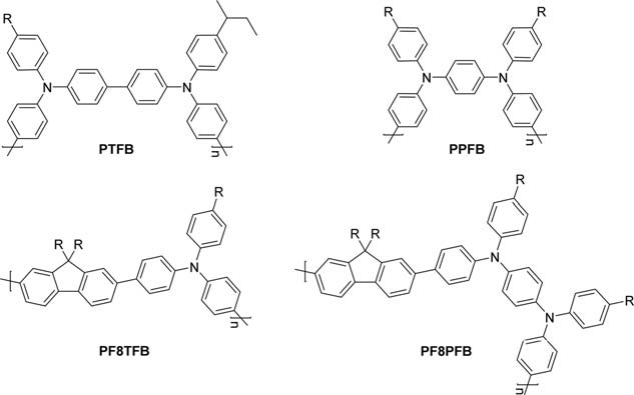
Structures of polymers synthesized in the Suzuki‐Miyaura cross‐coupling polymerizations (R = octyl).

The structures of the monomers used in this study are presented in Scheme 3, with the structures of the polymers produced presented in Scheme 4. The performance of the bis MIDA boronate ester monomers (e.g., **1b**) were compared against the equivalent triarylamine bis pinacol boronate esters in cross‐coupling reactions with the dibromo co‐monomer(s) (**3** and **4**).

## EXPERIMENTAL

### Materials

Monomers, **1a**, **2a**, **2c**, **3**, and **4** were kindly supplied by Cambridge Display Technology Ltd. Bifunctionalized BMIDA 4‐octyl phenyl diphenyl amine (TFB) and *N*, *N*′‐bis(4‐octylphenyl)‐*N*, *N*′‐diphenyl phenylenediamine (PFB) monomers, **1b** and **2b**, were synthesized using a published route.^22^ The procedures are outlined in the Supporting Information. ^1^H NMR spectra of these monomers are presented in Supporting Information Figures S1 and S2. Pd(OAc)_2_ was supplied by Acros Organics. The ligand, 2‐dicyclohexylphosphino‐2′,6′‐dimethoxybiphenyl (SPhos), potassium phosphate tribasic (K_3_PO_4_), 1,4‐dioxane, and toluene were all purchased from Aldrich Ltd. and used as supplied.

### NMR Studies of the Hydrolysis of Bis MIDA Monomer, 1b

A J. Young's NMR tube was charged under inert atmosphere with **1b**, (1.0 equiv., 14.0 mg, 0.021 mmol), mesitylene as internal reference (1.0 μL), and suspended in anhydrous d_8_‐tetrahydrofuran (d_8_‐THF) (0.6 mL). Subsequently, D_2_O (30.0 equiv. per BMIDA moiety), was added [**1b**] = 3.5 × 10^−2^ M, and the reaction mixture was vigorously shaken to homogenize before recording its NMR spectrum (*t*_0_). Then the tube was rotated at ambient temperature (10 rpm) or heated in an oil bath at 60 °C, and followed by NMR (^1^H and 11B) spectroscopy at different reaction times.

### Representative Polymerization Procedure (Entry 19)

Equimolar amounts of monomers, **1b** (66.74 mg, 0.1 mmol) and **3** (45.92 mg, 0.1 mmol), were placed into a Radley's carousel tube (Table 1). Small amounts of toluene (0.50 mL, 0.43 g) and dioxane (1.00 mL, 1.03 g) were next washed into the tube. Stock solutions of the monomers in solvents were not prepared owing to the poor solubility of the BMIDA monomers at room temperature in the desired solvents. A stock solution of K_3_PO_4_ was prepared (consisting of 0.1592 g, 0.8 mmol K_3_PO_4_ per 1 mL water). An amount of this base solution (1.1592 g) was transferred into the reaction tube. The tube contents were then stirred with a magnetic flea while being thoroughly degassed by bubbling nitrogen gas through the solution for 20 min. A stock solution of the palladium (II) acetate Pd(OAc)_2_/SPhos catalyst system in toluene was prepared, composed of: Pd(OAc)_2_ (3.36 mg, 0.015 mmol), SPhos (12.33 mg, 0.030 mmol), and toluene (1.50 mL, 1.30 g). The catalyst solution was stirred with a magnetic flea for about 20 min while being thoroughly degassed by three repeated cycles of evacuation followed by replenishment with nitrogen gas. The previously degassed reaction tube and contents were then placed into a carousel reactor maintained at 90 °C. After 10 min, a glass syringe was used to inject 0.5 mL of the catalyst solution into the now heated reaction tube contents while maintaining a nitrogen environment. The reaction tube was stirred under a nitrogen environment at 90 °C for a period of 24 h.

**Table 1:** Suzuki Cross‐coupling Polymerizations of Triarylamine Boronate Monomers (**1a, 1b, 2a, 2b**) with Dibromo Monomers (**2c, 3, 4**), Using Pd(OAc)_2_/SPhos Catalyst and K_3_PO_4_ as Base, in Mixed Solvent Systems^a^

Entry	Monomer M_1_	Monomer M_2_	Solvent Composition^b^ (T:D:W) (mL)	Catalyst Pd(OAc)_2_/SPhos (mol %)	Temperature (°C)	Time (h)	Polymer	Molar Mass^c^ (kg mol^−1^)		
								*M*_n_	*M*_w_	*Ð*
1^d^	**1a**	**3**	0: 5: 1	2.5/5.0	90	24	PTFB	17.9	28.5	1.6
2^d^	**1b**	**3**	0: 5: 1	2.5/5.0	90	24	PTFB	4.3	10.7	2.5
3^d^	**2a**	**2c**	0: 5: 1	2.5/5.0	90	24	PPFB	39.4	73.1	1.9
4^d^	**2b**	**2c**	0: 5: 1	2.5/5.0	90	24	PPFB	14.3	24.0	1.7
5^d^	**1a**	**4**	0: 4: 1	2.5/5.0	90	4	PF8TFB	14.8	32.5	2.2
6^d^	**1b**	**4**	0: 4: 1	2.5/5.0	90	4	PF8TFB	17.2	36.9	2.2
7^d^	**2a**	**4**	0: 4: 1	2.5/5.0	90	4	PF8PFB	14.9	40.5	2.7
8^d^	**2b**	**4**	0: 4: 1	2.5/5.0	90	4	PF8PFB	9.7	21.5	2.2
9^d^	**2a**	**2c**	1: 4: 1	2.5/5.0	90	5	PPFB	13.6	55.4	4.1
10^d^	**2b**	**2c**	1: 4: 1	2.5/5.0	90	5	PPFB	14.9	40.9	2.7
11	**1a**	**3**	3: 2: 1	2.5/5.0	90	24	PTFB	7.4	19.8	2.7
12	**1b**	**3**	3: 2: 1	2.5/5.0	90	24	PTFB	6.4	13.3	2.1
13	**1b**	**3**	3: 2: 1	2.5/5.0	60	24	PTFB	6.4	11.0	1.7
14	**1a**	**3**	3: 1: 1	5.0/10.0^b^	90	48	PTFB	11.5	32.1	2.8
15	**1b**	**3**	3: 1: 1	5.0/10.0^b^	90	24	PTFB	5.1	8.8	1.7
16	**1a**	**3**	1: 1: 1	2.5/5.0	80	24	PTFB	10.2	37.8	3.7
17	**1b**	**3**	1: 1: 1	2.5/5.0	80	24	PTFB	6.9	17.0	2.5
18	**1a**	**3**	1: 1: 1	2.5/5.0	90	24	PTFB	13.7	30.6	2.2
19	**1b**	**3**	1: 1: 1	2.5/5.0	90	24	PTFB	9.7	21.9	2.3
20	**1a**	**4**	1: 1: 1	2.5/5.0	90	24	PF8TFB	32.3	76.8	2.4
21	**1b**	**4**	1: 1: 1	2.5/5.0	90	24	PF8TFB	20.4	61.5	3.0
22	**2a**	**4**	1: 1: 1	2.5/5.0	90	24	PF8PFB	28.4	93.5	3.3
23	**2b**	**4**	1: 1: 1	2.5/5.0	90	24	PF8PFB	20.0	59.4	3.0

a Reaction conditions: equimolar quantities of monomers, **M_1_** + **M_2_**, total monomer = 0.2 mmol.

b Dissolved in mixed solvent system, where T is toluene, D is dioxane, and W is H_2_O (mL); 4 equivalent mmol of K_3_PO_4_ (8 equiv. entries 14 and 15).

c Molar mass determined by GPC in THF versus polystyrene standards.

d Reactions in which the polymer precipitated out of the solvent mixture during the reaction time. Large amounts of polymeric material precipitated out of most of the dioxane rich PTFB and PPFB reactions (entries 1–4, 9) within 10 min of start of reaction. Only Entry 10 reaction remained homogeneous for a few hours. GPC analysis of these entries refers to the molar mass of the precipitated polymer recovered after the reaction time. The remaining GPC analysis of entries 5–8, 11–23 refers to molar masses of all polymeric material (both precipitated and soluble) recovered after the respective reaction times.

### Characterization of the Reaction Products (Entry 19)

A sample of the reaction mixture (0.5 mL) was removed after 5 h under a nitrogen atmosphere using a glass syringe and placed into a small amount of toluene (1.0 mL) (Table 1). The sample solution was allowed to cool, before being added drop wise to a stirred excess amount of chilled methanol (5 mL) to precipitate the polymer. The polymer formed a fine dispersion in the stirred methanol. A pipette was used to transfer samples of this dispersal into a pair of vials suitable for use in a centrifuge. The vial samples were placed in a centrifuge at 14,000 rpm for 10 min. The supernatant layer was removed from above the separated polymer. The polymer samples were then dried and redissolved in THF for GPC analysis. After 24 h, upon completion of the reaction, the remaining tube contents were added to a small amount of toluene (2.0 mL). The diluted reaction solution was then allowed to cool, before being added drop wise to a stirred excess amount of chilled methanol (20 mL) to precipitate the polymer.

Samples of the dispersion were then treated similarly to prepare samples for GPC analysis. Polymer molar mass and molar mass distribution was determined by GPC in THF solution using a Viscotek GPCmax VE2001 and a Viscotek VE3580 RI detector (referenced to polystyrene standards). The sample solutions were made up in THF (1 mg polymer per mL solvent) and filtered before injection. Matrix‐assisted laser desorption/ionization time‐of‐flight (MALDI‐TOF) mass spectrometry was carried out using a Shimadzu Biotech AXIMA Confidence MALDI mass spectrometer in reflectron mode or linear (positive) mode, referencing against either PEG 1K or PPG 4K. Polymer samples where dissolved in THF to a concentration of 10 mg mL^−1^. A similar solution was prepared of the matrix (dithranol). Polymer sample solution of 1 µL was mixed with 10 µL of matrix solution. The solution was mixed and approximately 0.5 µL was spotted on to the sample plate. ^1^H NMR spectra of the copolymers recovered in each case were recorded with a Bruker 400 MHz NMR instrument in deuterated chloroform (CDCl_3_) using tetramethylsilane as an internal standard (examples are presented in Supporting Information Figs. S3–S6).

## RESULTS AND DISCUSSION

### Hydrolysis of Bis MIDA Monomer, 1b

**Scheme 5:**
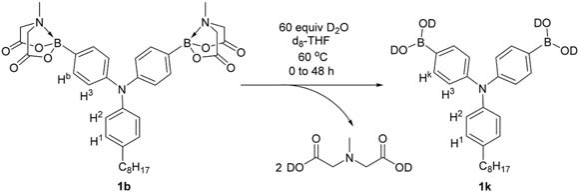
Conditions used in hydrolysis studies of bis MIDA monomer, **1b**.

**Figure 1:**
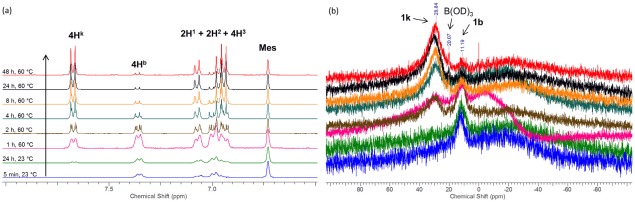
(a) Collated aryl proton regions of ^1^H NMR spectra in *d*_8_‐THF of the hydrolysis of **1b** to **1k**. Reaction conditions as described in Scheme 5. Mesitylene (Mes) was added as internal standard. (b) Collated 11B NMR spectra in *d*_8_‐THF of the hydrolysis of **1b** to **1k**. Reaction conditions as described in Scheme 5. [Color figure can be viewed at wileyonlinelibrary.com]

Hydrolysis of the BMIDA moiety by water under neutral conditions has been observed for a range of aryl‐ and heteroaryl‐BMIDA boronate esters.^23^, ^26^ To understand the species involved in the Suzuki‐Miyaura reaction, the hydrolysis of monomer **1b** was studied in THF/water mixtures. The respective boronic acid (Scheme 5) **1k** is formed with the MIDA diacid precipitating in each case under these conditions. The bis MIDA monomer **1b** is not completely soluble in THF at RT or 60 °C at the initial concentration used in the polymerization. However, the formed boronic acid, **1k**, is soluble in THF. As the hydrolysis to the boronic acid proceeded at 60 °C, eventually all of **1b** present dissolved in the solution. This occurred after 2 h in the *in situ* ^1^H NMR experiment [Fig. 1(a)], as from this point onwards the combined integral area of resonances (between *δ* = 6.9–7.1 ppm) attributed to the 8 aryl protons (2H^1^ + 2H^2^ + 4H^3^) that are unchanged in the hydrolysis remains constant relative to integral area of the internal standard (mesitylene) peak at 6.7 ppm. The extent of hydrolysis was determined from comparing the integral area of the aryl hydrogens unaffected by hydrolysis (*δ* = 6.9–7.1 ppm) against those signals associated with the four aryl protons (4H^k^) directly next to the B(OD)_2_ groupings in **1k** (*δ* = 7.7 ppm). Compound **1b** is hydrolyzed by 48% and 85% after 2 and 8 h, respectively. There is no evidence of protodeboronation of **1b** or **1k** (by ^1^H NMR spectroscopy). Boric acid (*δ* 11B = 20.1) is observed as a very minor by‐product [Fig. 1(b)]. No significant changes observed from 24 to 48 h of reaction, where **1b** is almost fully consumed.

### Polymerizations in Dioxane/Water Mixtures

**Figure 2:**
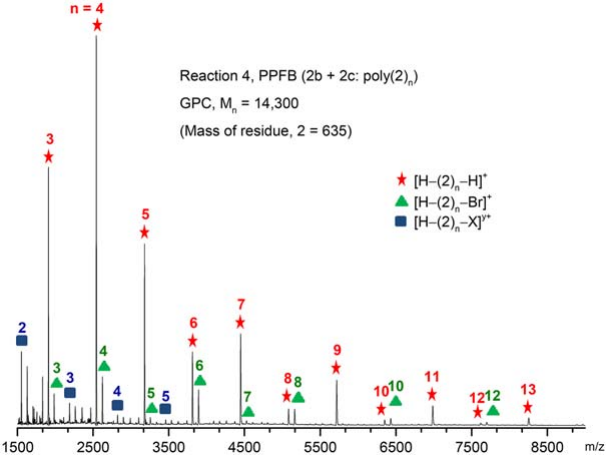
MALDI‐TOF mass spectrum of precipitated PPFB polymer recovered from cross‐coupling reaction of bis BMIDA PFB monomer (**2b**) with dibromo PFB monomer (**2c**) (Table 1, entry 4). [Color figure can be viewed at wileyonlinelibrary.com]

Initial Suzuki‐Miyaura cross‐coupling polymerizations of the pinacol boronate ester monomers (**1a**, **2a**) and the MIDA boronate ester monomers (**1b**, **2b**) with their respective dibromo co‐monomers (**2c**, **3**) to produce PTFB and PPFB (Table 1, entries 1–4) were performed in dioxane/water mixtures similar to those outlined by Burke and coworkers.^6^ The BMIDA monomers proved to be only partially soluble in dioxane at room temperature, with the reactions initially taking on the appearance of an emulsion. However, all the boronate monomers proved to react very rapidly with their respective dibromo comonomers. For example, the reaction between PFB MIDA boronate ester monomer, **2b**, and dibromo PFB monomer, **2c**, (Table 1, entry 4), in dioxane/water solvent mixture (5: 1 mL) at 90 °C, proceeded with PFB polymer precipitating out of the solvent mixture within 10 min (similar timescales observed for reactions 1–3). The precipitated PPFB material was recovered from the solution, redissolved in hot toluene and reprecipitated in excess methanol. GPC analysis of this sample indicated that polymer of number average molar mass (*M*_n_), *M*_n_ = 14,300, had been formed before precipitation. The molar mass distributions of polymer in the remaining reaction solution were sampled after 24 h and 48 h. A narrower molar mass distribution of polymer in solution was evident after 48 h (*Ð* = 1.6) owing to the longer growing polymer chains having by this stage precipitated out of solution, leaving predominantly inactive oligomers (*M*_n_ ∼ 5,000) still in solution. The MALDI‐TOF mass spectrum of the precipitated PPFB polymer recovered from reaction 4 is presented in Figure 2, with assignment details outlined in Supporting Information Table S1. The most significant distribution of mass peaks can be attributed to PPFB terminated on both ends by hydrogen atoms, that is, [H–(2)_n_–H]^+^, with structures equating to at least *n* = 15 evident. The next most significant series can be attributed to PPFB end terminated with one bromine and one hydrogen atom, that is, [H–(2)_n_–Br]^+^, with structures equating at least *n* = 14 evident. The insolubility of the higher molecular weight PTFB and PPFB polymers in these dioxane/water mixtures (Table 1, entries 1–4) results in premature precipitation of the polymers before the reaction is complete.

**Figure 3:**
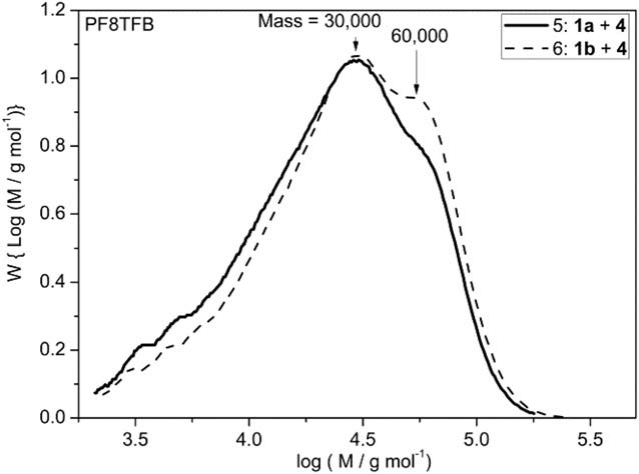
GPC molar mass distributions of PF8TFB polymers obtained from cross‐coupling reactions of pinacol ester or MIDA boronate ester TFB monomers (**1a** or **1b**) with dibromo F8 monomer (**4**) in dioxane/water, 4: 1 (mL) solvent mixtures at 90 °C after 4 h (Table 1, entries 5 and 6).

Suzuki‐Miyaura cross‐coupling reactions of the TFB or PFB monomers (**1**, **2**) with the dibromo fluorene monomer, **4**, gave poly (9,9‐dioctylfluorene [F8] ‐alt‐ 4‐octylphenyl diphenyl amine [TFB]) (PF8TFB) and poly(9,9‐dioctylfluorene [F8] ‐alt‐ N,N′‐bis(4‐octylphenyl)‐N,N′‐diphenyl phenylenediamine) [PFB] (PF8PFB). The solvent composition initially used was dioxane/water, 4:1 (mL) and the reaction is conducted at 90 °C (Table 1, entries 5–8). Copolymerizations with the dibromo fluorene monomer, **4**, improved the solubility of the polymers in the dioxane/water mixtures, with the respective polymers only precipitating out of solution after 4 h. The overall *M*_n_ of all PF8TFB material collected from reactions with pinacol ester and BMIDA functionalized monomers (Table 1, entries 5 and 6) were 14,800 and 17,200, respectively, after this short reaction time. This single phase system yielded polymers exhibiting very similar molar mass distributions regardless of whether the starting boronate monomer was either pinacol or BMIDA based (*Ð* = 2.2). A closer comparison of the molar mass distributions of the polymers recovered from the two reactions clearly indicates that the BMIDA reaction (Table 1, entry 6) is marginally shifted toward higher molar mass material, with a new peak emerging equating to a molar mass of 60,000, as outlined in Figure 3. The doubling in molar mass suggests late stage chain coupling as previously observed in the synthesis of P3HT from BMIDA monomers.^16^

### Polymerizations in Biphasic Conditions with Toluene

**Figure 4:**
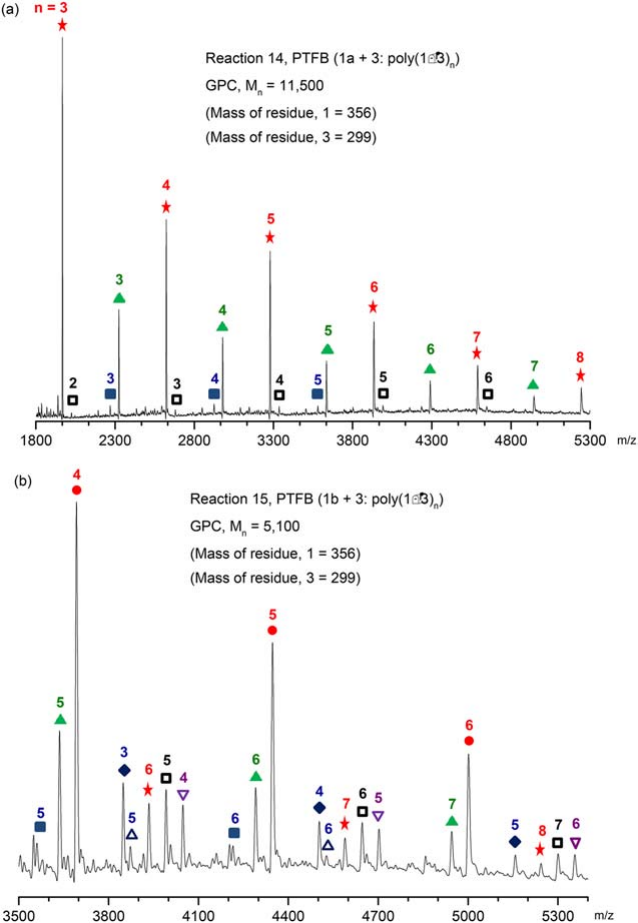
MALDI‐TOF mass spectra of PTFB polymers recovered from cross‐coupling reactions of **(a)** bis pinacol ester TFB monomer (**1a**) with dibromo TFB monomer (**3**) (Table 1, entry 14) and **(b)** bis BMIDA TFB monomer (**1b**) with dibromo TFB monomer (**3**) (Table 1, entry 15). [Color figure can be viewed at wileyonlinelibrary.com]

Toluene was added to the solvent mixtures to maintain the solubility of the polymers during the course of the reaction. Reactions were conducted between PPFB monomers, **2a** or **2b** with **2c**, in solvent mixtures that included 1 mL of toluene at the expense of the dioxane content. The reaction with the PFB pinacol ester again resulted in rapid precipitation of polymer (Table 1, entry 9), whereas the reaction with the MIDA boronate ester remained homogeneous for a few hours, before material precipitated out of solution (Table 1, entry 10). Polymers of intermediate molecular weights (*M*_n_ = 14,000–15,000) were obtained after 5 h in each case, with broader mass distributions than those prepared in toluene free reactions (Table 1, entries 3 and 4). The proportion of toluene present in the solvent mixtures was further increased to toluene (T)/dioxane (D)/water (W), 3:2:1 (mL) and PTFB polymers (Table 1, entries 11 and 12) prepared by reactions at 90 °C for 24 h. The polymers remained in solution throughout the reaction but were recovered with low *M*_n_ = 6,000–7,000. The GPC trace of the polymer from reaction 12 also indicated conversion to polymer of 96%. A similar reaction with the bis MIDA boronate ester completed at 60 °C (Table 1, entry 13) produced polymer of similar molar mass (*M*_n_ = 6,000) with a lower *Ð* of 1.7, while showing only low overall conversion to polymer of 66% (Supporting Information Fig. S7). This suggests that the two phase solvent reactions are not consistent with a conventional step‐growth polymerization. The low conversion to polymer evident after 24 h at 60 °C indicated that, even in presence of base, hydrolysis and cross‐coupling of the bis MIDA monomer under these biphasic conditions was relatively slow; hence, all future reactions were completed at higher temperatures. Lower molar mass polymer, *M*_n_ = 5,000 was obtained from reactions completed in a T/D/W, 3:1:1 (mL) solvent system at 90 °C (Table 1, entry 15), even using double the amount of catalyst (5 mol %), ligand (10 mol %), and base (8 equivalents). The cross‐coupling reaction of the bis pinacol boronate ester TFB monomer (**1a**) with the dibromo monomer (**3**) in a similar solvent mixture at 90 °C (Table 1, entry 14) yielded polymer of higher molar mass, *M*_n_ = 11,500. The MALDI‐TOF mass spectrum of PFTB polymer produced from the cross‐coupling reaction of the bis pinacol boronate ester FTB monomer (**1a**) with dibromo FTB monomer (**3**) (reaction 14) is presented in Figure 4(a), with assignment details outlined in Supporting Information Table S2. The FTB residue masses for 1 and 3 repeat units are 356 and 299 mass units, respectively. The main series (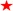) are associated with even numbered residue PFTB chain structures terminated at both ends by hydrogen atoms, that is, [H–(**1** – **3**)_n_–H]^+^ (Table 2). The two next most significant series are attributed to odd numbered residue polymers in which both ends are terminated with the same monomer residues. The most prominent of these two series (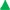) can be attributed to structures containing residues of monomer **1** next to ends terminated with hydrogen atoms, that is, [H–(**1** – **3**)_n_–**1** – H]^+^. The other series (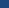) can be attributed to the equivalent for **3**, that is, [H – **3**–(**1** – **3**)_n_–H]^+^. The MALDI‐TOF mass spectrum of PFTB produced from the reaction of bis BMIDA TFB monomer (**1b**) with **3** (reaction 15) is presented in Figure 4(b), with assignment details outlined in Supporting Information Table S3. The predominant series of peaks (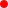) would appear to equate to PTFB structures rich in residues of **1**, for example, [H–(**1**)_3_–(**1** – **3**)_n_–H]^+^. The remaining distribution series are present in similar smaller amounts, with four more also associated with polymer structures containing excess residue **1** than would be expected in a classical cross coupling polymerization, either [H–(**1**)_*x*_–(**1** – **3**)_*n*_–H]^+^, where *x* = 1, 2, 4 (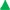, 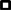, 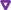), or a doubly bromine terminated version of the main peak distribution, defined as [Br – **3**–(**1**)_4_–(**1** – **3**)_n_–Br]^+^ (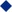). The prominence of these polymeric structures in the material obtained from cross‐coupling reaction with the BMIDA FTB monomer (**1b**) indicates that homocoupling of boron end groups resulting from the BMIDA monomer is competing with the cross‐coupling reaction resulting in a reduction in the molar mass of PFTB polymer obtained (*M*_n_ = 5,100, Table 1, entry 15), relative to a similar reaction with the pinacol boronate ester FTB monomer (**1a**) (*M*_n_ = 11,500, Table 1, entry 14). A significant amount of homocoupling, originating from boronate ester monomers used in Suzuki polycondensations of P(Cbz‐*alt*‐TBT) and PCDTBT, has recently been observed.^27^, ^28^ A difference in either reactivity or solubility of the two boronate ester monomers (**1a** and **1b**) in these multiphase solvent mixtures may also contribute to changes in the relative rates of homo‐ versus heterocoupling in these particular reactions. As we discussed earlier, in some single phase (dioxane + water) reactions, there was little difference in the polymer produced from either boronate monomer in comparable reactions (Table 1, entries 5 and 6). These results indicated that while adding toluene to the solvent mixture aided polymer solubility during the course of the reaction, creating a new phase had a detrimental effect on the molar mass of polymers obtained from MIDA boronate ester monomers, which could not be arrested by increasing the amount of the catalyst system or partially reducing the overall reaction volume.

**Table 2:** PTFB Polymer Structures Assigned from MALDI Mass Spectra of Polymers Recovered from Cross‐coupling Reactions of Pinacol Ester TFB (**1a**) or BMIDA (**1b**) Monomer with Dibromo TFB Monomer (**3**) (Table 1, Entries 14 and 15) [Color table can be viewed at wileyonlinelibrary.com]

	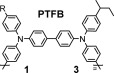	Main Mass Peaks Evident in MALDI‐TOF Spectra (Residue 1 Mass = 356, Residue 3 Mass = 299) (R = octyl)
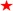	[H–(1–3)_n_–H]^+^	3279, 3935, 4589, 5244
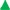	[H–(1–3)_n_–1–H]^+^	2980, 3635, 4290, 4945
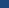	[H–3–(1–3)_n_–H]^+^	2269, 2925, 3581
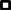	[H–(1)_2_–(1–3)_n_–H]^+^	3336, 3992, 4646, 5300
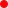	[H–(1)_3_–(1–3)_n_–H]^+^	3692, 4347, 5002
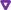	[H–(1)_4_–(1–3)_n_–H]^+^	4048, 4702, 5357
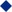	[Br–3–(1)_4_–(1–3)_n_–Br]^+^	3849, 4504, 5157
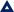	[H–(3)_2_–(1–3)_n_–H]^+^	3872, 4529

**Figure 5:**
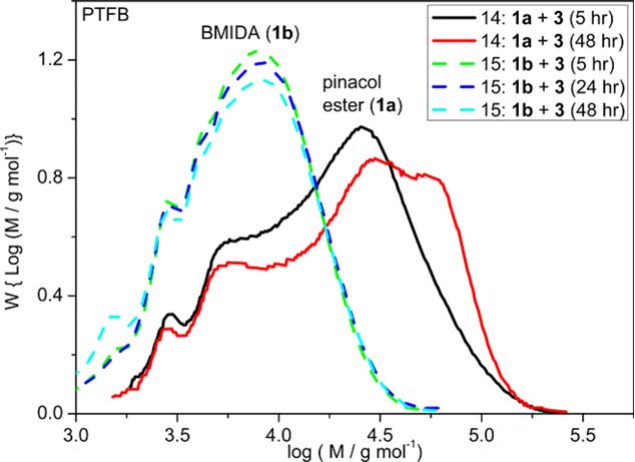
GPC molar mass distributions of PTFB polymers obtained from cross‐coupling reactions of bis pinacol ester or bis BMIDA functionalized TFB monomers (**1a** or **1b**, respectively) with the dibromo TFB monomer (**3**) in T:D:W, 3:1:1 (mL), solvent mixtures at 90 °C (Table 1, entries 14 and 15) after different time intervals. [Color figure can be viewed at wileyonlinelibrary.com]

The molar mass distributions of polymers obtained from cross‐coupling reactions of the pinacol boronate ester monomer (**1a**) or BMIDA monomer (**1b**) with dibromo FTB monomer (**3**) in directly comparable reactions (Table 1, entries 14 and 15) at 90 °C are outlined in Figure 5. The reaction progress with time was also monitored, including at 80 °C (Table 1, entries 16 and 19; Supporting Information Fig. S8). In each instance, the polymer chains created in reactions starting with a BMIDA‐based monomer showed no signs of cross‐coupling further to create longer chains of higher molar mass beyond 5 h.

The step growth polymerizations involving the pinacol boronate ester monomer still contained active chain ends after 5 h that reacted further to create longer chains by GPC after 24 to 48 h. In one particular reaction containing a BMIDA monomer (Table 1, entry 19), a second batch of catalyst/ligand dissolved in toluene (similar to initial charge) was added to the reaction mixture after 5 h to see whether this would induce further cross‐coupling between the existing polymer chains. No change in molar mass distribution of polymer was observed even after a total reaction time of 24 h.

**Figure 6:**
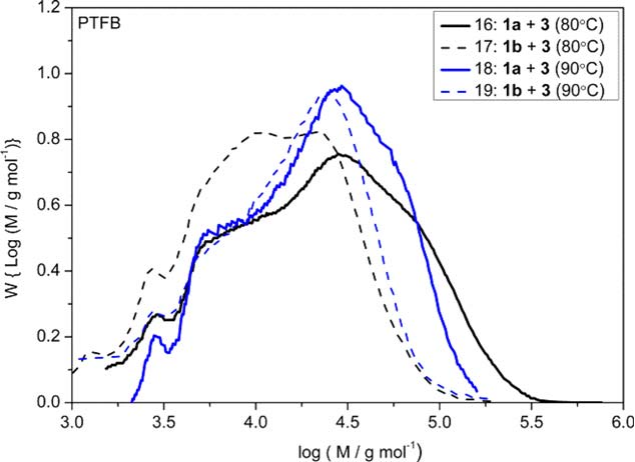
GPC molar mass distributions of PTFB polymers obtained from cross‐coupling reactions of pinacol ester or BMIDA functionalized TFB monomers (**1a** or **1b**, respectively) with the dibromo TFB monomer (**3**) in T:D:W, 1:1:1 (mL), solvent mixtures at 80 °C and 90 °C (Table 1, entries 16–19). [Color figure can be viewed at wileyonlinelibrary.com]

**Figure 7:**
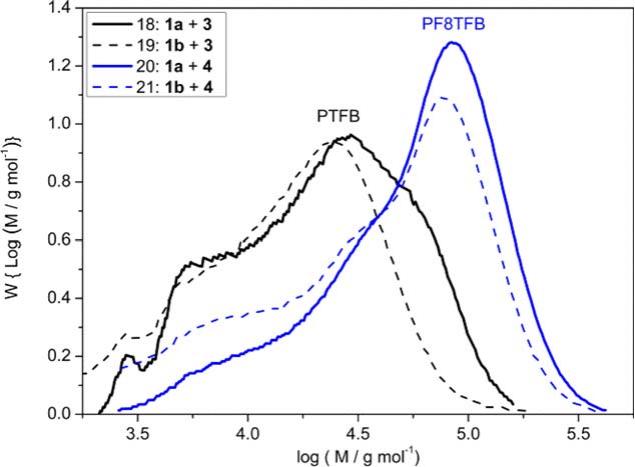
GPC molar mass distributions of polymers obtained from cross‐coupling reactions of pinacol ester or BMIDA monomers (**1a** or **1b**) with dibromo TFB or F8 monomers (**3** or **4**) in T:D:W, 1:1:1 (mL), solvent mixture at 90 °C after 24 h (Table 1, entries 18–21). [Color figure can be viewed at wileyonlinelibrary.com].

Cross‐coupling of the TFB boronate ester monomers (**1a** or **1b**) with dibromo monomer **3** in a T/D/W, 1:1:1 mL at 90 °C (Table 1, entries 18, 19) gave a marked increase in the molar mass of polymer, with polymers of *M*_n_ = 13,700 (*Ð* = 2.2) produced from the cross‐coupling reaction of monomer, **1a**, with dibromo monomer, **3** (Table 1, entry 18). As before, higher molar mass PFTB polymers were produced in the cross‐coupling reactions of the TFB pinacol boronate ester monomer, **1a**, with the dibromo monomer, **3**, than were achieved with the BMIDA TFB monomer, **1b**, in comparable multiphase reactions at 80 and 90 °C (Fig. 6). Reactions with the dibromo fluorene monomer (**4**) were also completed in T:D:W, 1:1:1 (mL) solvent mixtures (Table 1, entries 20–23 and Fig. 7). The inclusion of the dibromo F8 monomer (**4**) as a co‐monomer, instead of dibromo FTB monomer (**3**), increased the molar masses of polymers obtained in the reactions with both the pinacol boronate ester and BMIDA monomers: peak molar masses (*M*_p_) of the polymers obtained in reactions with **1a** increased from 30,000 to 90,000, and in reactions with **1b** from 25,000 to 84,000. Cross‐coupling reactions of the MIDA boronate ester TFB or PFB monomer (**1b** or **2b**) with dibromo F8 monomer (**4**) (Table 1, entries 21 and 23) both yielded polymers with *M*_n_ of approximately 20,000. A scale‐up reaction of entry 23 produced PF8PFB in yield of 0.98 g (80%) exhibiting similar molecular weight (experimental details in Supporting Information Table S4 and Fig. S9).

## CONCLUSIONS

Suzuki‐Miyaura cross‐coupling reactions of bis pinacol boronate ester or bis MIDA boronate ester monomers, with dibromo comonomers, produced high molar mass conjugated polymers after optimization of the reaction conditions. It appears that polymer chains, generated from cross‐coupling reactions of bis BMIDA monomer (**1b**) with dibromo comonomer (**3**) at 90 °C, stop growing within a reaction time of 5 h using this catalyst system in biphasic solvent mixtures. MALDI‐TOF mass spectral evidence suggest homocoupling of residues associated with the MIDA boronate ester monomer is occurring which could contribute to limiting the achievable molar mass of polymer.

Cross‐coupling reactions undertaken in dioxane + water mixtures resulted in rapid precipitation of the (co)polymer often before the reaction had reached maximum molar mass or high conversion. However, the BMIDA monomer (**1b**) proved as successful as the pinacol ester (**1a**) in these single phase copolymerizations with dibromo fluorene monomer (**4**) to produce PF8TFB. A polymer of high molar mass, *M*_n_ = 17,000 (*Ð* = 2.2) was precipitated from the solution within only 4 h. Optimum reaction conditions for maintaining the polymer in solution, to achieve higher molar mass, were achieved in T:D:W (1:1:1) solvent mixtures in biphasic reactions. PF8TFB and PF8PFB polymers (approximately *M*_n_ = 20,000) with broad molar mass distributions, *Ð* = 3.0, were obtained from reactions starting with the respective BMIDA monomers in this solvent mixture after 24 h (Table 1, entries 21 and 23).

## Supporting information

Supporting Information
