# Supplementary material for: Use of N‐methyliminodiacetic acid boronate esters in suzuki‐miyaura cross‐coupling polymerizations of triarylamine and fluorene monomers
Source: J Polym Sci A Polym Chem. 2017 Jul 3;55(17):2798–806. doi: 10.1002/pola.28682 (PMC5600090; doi:10.1002/pola.28682)
Supplement: Supplementary file 1 — Supporting Information [file POLA-55-2798-s001.doc]

**SUPPORTING INFORMATION**

**Use of N-methyliminodiacetic acid boronate esters in suzuki-miyaura cross-coupling polymerizations of triarylamine and fluorene monomers**

Andrew B. Foster^1,*^, Viktor Bagutski^1^, Josue I. Ayuso-Carrillo^1^, Martin Humphries^2^, Michael J. Ingleson^1^, Michael L. Turner^1,*^

^1^School of Chemistry, University of Manchester, Oxford Road, Manchester M13 9PL, UK

^2^Cambridge Display Technology Ltd., Unit 12 Cardinal Park, Cardinal Way, Godmanchester, Cambridgeshire PE29 2XG, United Kingdom

Corresponding authors:

* University of Manchester. Oxford Road, Manchester M13 9PL, UK. Emails: [michael.turner@manchester.ac.uk](mailto:michael.turner@manchester.ac.uk) & [andrew.foster@manchester.ac.uk](mailto:andrew.foster@manchester.ac.uk); Fax: +44(0)1612754273; Tel: +44(0)1612754625.

| **Materials and experimental comments** | **3** |
| --- | --- |
| **Preparation of monomers** | **3** |
| Synthesis of TFB(BMIDA)_2_, **1b** | **3** |
| **Figure S1.** ^1^H NMR of TFB(BMIDA)_2_ in deuterated acetone. | **5** |
| Synthesis of PFB(BMIDA)_2_, **2b** | **6** |
| **Figure S2.** ^1^H NMR of PFB(BMIDA)_2_ in deuterated acetone. | **8** |
| **^1^H NMR analysis of copolymers, PTB, PPFB, PF8TFB and PF8PFB** | **9** |
| **Figure S3**. ^1^H NMR spectra of PTFB produced in (a) Reaction 14 (**1a** + **3**: poly(**1**-**3**)_n_) and (b) Reaction 15 (**1b** + **3**: poly(**1**-**3**)_n_) in CDCl_3_ | **10** |
| **Figure S4.** ^1^H NMR spectra of PPFB produced in (a) Reaction 9 (**2a** + **2c**: poly(**2**)_n_) and (b) Reaction 10 (**2b** + **2c**: poly(**2**)_n_) in CDCl_3_ | **11** |
| **Figure S5.** ^1^H NMR spectra of PF8TFB produced in (a) Reaction 5 (**1a** + **4**: poly(**1**-**4**)_n_) and (b) Reaction 6 (**1b** + **4**: poly(**1**-**4**)_n_) in CDCl_3_ | **12** |
| **Figure S6.** ^1^H NMR spectra of PF8PFB produced in (a) Reaction 7 (**2a** + **4**: poly(**2-4**)_n_) and (b) Reaction 8 (**2b** + **4**: poly(**2-4**)_n_) in CDCl_3_ | **13** |
| **Further GPC analysis of copolymers** | **14** |
| **Figure S7.** GPC chromatograms of polymers obtained in cross-coupling reactions of bis BMIDA TFB monomer (**1b**) with dibromo TFB monomer (**3**) after 24 hours at 60 and 90 °C respectively (reactions 13 & 12). The conversion percentages are calculated from the respective integral areas of peaks associated with the monomer (TFB) and the polymer (PTFB). | **14** |
| **Figure S8.** GPC molar mass distributions of PTFB polymers obtained from cross-coupling reactions of bis pinacol ester or bis BMIDA functionalized TFB monomer (**1a** or **1b**) with dibromo TFB monomer (**3**) in toluene / dioxane / water, 1: 1: 1 (ml) solvent mixtures at 80 or 90 °C respectively, after different time intervals (reactions 16 & 19). | **14** |
| **MALDI analysis of copolymers** | **15** |
| **Table S1.** Expected (Exp.) and Actual (Act.) MALDI peak assignments of PPFB (Figure 2). | **15** |
| **Table S2.** Expected (Exp.) and Actual (Act.) MALDI peak assignments of PTFB (Figure 4(a)). | **15** |
| **Table S3.** Expected (Exp.) and Actual (Act.) MALDI peak assignments of PFTB (Figure 4(b)). | **16** |
| **Scale-up PF8PFB reaction (larger scale version of reaction 23)** | **17** |
| **Table S4.** GPC analysis of the molar masses of polymers obtained from Suzuki cross-coupling reactions of **2b** and **4** in toluene / dioxane / water (1: 1: 1 ml) solvent mixtures in presence of Pd(OAc)_2_ / SPhos (2.5 / 5.0 mol %) catalyst system at 90 °C after 24 hr. | **19** |
| **Figure S9.** Molar mass distributions of PF8PFB polymers obtained in cross-coupling reactions of **2b** with **4** at 90 °C after 24 hours (reactions 23 & 23 scale-up). | **19** |
| **References** | **19** |

**Materials and experimental comments**

All materials were used as received from commercial vendors, except for the bis-TMS ester of N-methyliminodiacetic acid (termed bis-TMS-MIDA), the preparation of which is described in literature.[^1^](#_ENREF_1) All borylation reactions were performed using standard Schlenk line techniques and anhydrous solvents. BCl_3_ purchased as a 1M solution in CH_2_Cl_2_ or heptanes was found to be of variable molarity. Therefore an excess of reagents can be used to ensure sufficient borylating agent is present for full borylation. For large scale where this is not economical and reactions where product distribution was sensitive to stoichiometry the molarity of BCl_3_ solutions was approximately quantified by titration with PPh_3_ (using ^11^B and ^31^P NMR spectroscopy). Alternatively equimolar Me_2_NTol and BCl_3_ (in heptanes) combined in hexanes results in precipitation of the adduct (Me_2_NTol)BCl_3_ as a colourless solid which can then be readily isolated and used to obtain exact stoichiometries. Furthermore (Me_2_NTol)BCl_3_ can be handled for short periods in air. Reactions of AlCl_3_ with amines in DCM as well as RBCl_2_ with DMI are both slightly exothermic, therefore stirring and cooling with ice water during addition and for 15 min afterwards is sufficient to keep the reaction under control. The same principle is applied in the procedure for preparation of MIDA-TMS_2_. Cooling with ice water is used during addition and first 0.5 h of the reaction. This is enough to keep the reaction temperature below 40 °C.

**Preparation of Monomers**

**Synthesis of TFB(BMIDA)_2_, 1b**

Part 1: To a stirred suspension of anhydrous AlCl_3_ (10.7 g, 80 mmol) in anhydrous dichloromethane (82 mL) *N*,*N*-dimethylaniline, termed DMA, (10.2 mL, 80 mmol) and BCl_3_ (100 mL of 0.8 m solution in dichloromethane, 80 mmol) were successively added at 0–5 °C (ice-water bath, this cooling is sufficient to keep the reaction below 40^o^C) under argon atmosphere (**CAUTION** mildly exothermic reaction cooling required, particularly if preparing on a large scale). The cooling bath was then removed and the reaction mixture was stirred at ambient temperature until complete homogenisation (~1 h). Then, 4-octyltriphenylamine (**TFB**) (13 g, 36.4 mmol) was added all at once and the reaction mixture was stirred at ambient temperature for another 2-24 h to accomplish formation of a bis-dichloroborane. Then, it was trans-esterified into MIDA-boronate (**TFB(BMIDA)_2_, 1b**) as follows.

Part 2: The mixture from Part 1 was diluted with anhydrous dichloromethane (250 mL), chilled to 0–5 °C and *N*,*N'*-dimethyl-1,3-imidazolin-2-one, DMI (17.2 mL, 160 mmol) (**CAUTION** mildly exothermic reaction cooling required, particularly if preparing on a large scale) and bis-TMS-MIDA (23.3 g, 80 mmol) were successively added by cannula with vigorous stirring. The cooling was removed and the reaction mixture was allowed to warm up to room temperature. After 24 h, the supernatant solution was filtered in an inert atmosphere and the residue was washed with dry dichloromethane (3×300 mL). The crude product was dried in vacuo to remove residual dichloromethane. Then, it was dissolved in hot acetone and passed through thin plug of celite. The filtrate was concentrated and left in a dark place for crystallisation. Filtration, washing with cold acetone and drying *in vacuo* (0.02 mbar, 24 h) afforded pure product as off-white free-flowing powder.

**TFB(BMIDA)_2_** can be prepared on a 1g or 15g scale using Part 1. No changes were made to the procedures (apart from scaling solvent volumes) on increasing the reaction scale.

According to Part 1, 4-octyltriphenylamine (**TFB**) (13 g, 36.4 mmol) afforded 14.6 g (60%) of pure product.

**^1^H NMR** (400 MHz, acetone-*d*_6_, δ, ppm): 7.44–7.40 (m, 4 H), 7.18–7.14 (m, 2 H), 7.04–7.00 (m, 6 H), 4.33 (d, ^2^*J*_(H,H)_ = 16.9 Hz, 4 H), 4.13 (d, ^2^*J*_(H,H)_ = 16.9 Hz, 4 H), 2.79 (s, 6 H, C*H_3_*N), 2.59 (dd, ^3^*J*_(H,H)_ = 8.1, 7.3 Hz, 2 H, C*H_2_*-Ar), 1.66–1.58 (m, 2 H, C*H_2_*CH_2_-Ar), 1.39–1.25 (m, 10 H), 0.88 (t, ^3^*J*_(H,H)_ = 6.9 Hz, 3 H, CH_3_)

**^13^C NMR** (100.6 MHz, acetone-*d*_6_, δ, ppm): 169.3 (4 C_quat_), 149.5 (2 C_quat_), 146.0 (C_quat_), 139.1 (C_quat_), 134.4 (4 CH), 130.2 (2 CH), 126.1 (2 CH), 123.4 (4 CH), 62.6 (4 CH_2_), 48.2 (2 CH_3_N), 35.9 (CH_2_-Ar), 32.6 (CH_2_), 32.4 (CH_2_), 30.2 (CH_2_), 30.1 (CH_2_), 30.0 (CH_2_), 23.3 (CH_2_), 14.4 (CH_3_)

**^11^B NMR** (128.4 MHz, acetone-*d*_6_, δ, ppm): 11.9 (s)

**HRMS (ESI)**: calcd. for C_36_H_42_B_2_N_3_O_8_^–^ [M + Cl^–^] 702.2930, found: 702.2895.

**
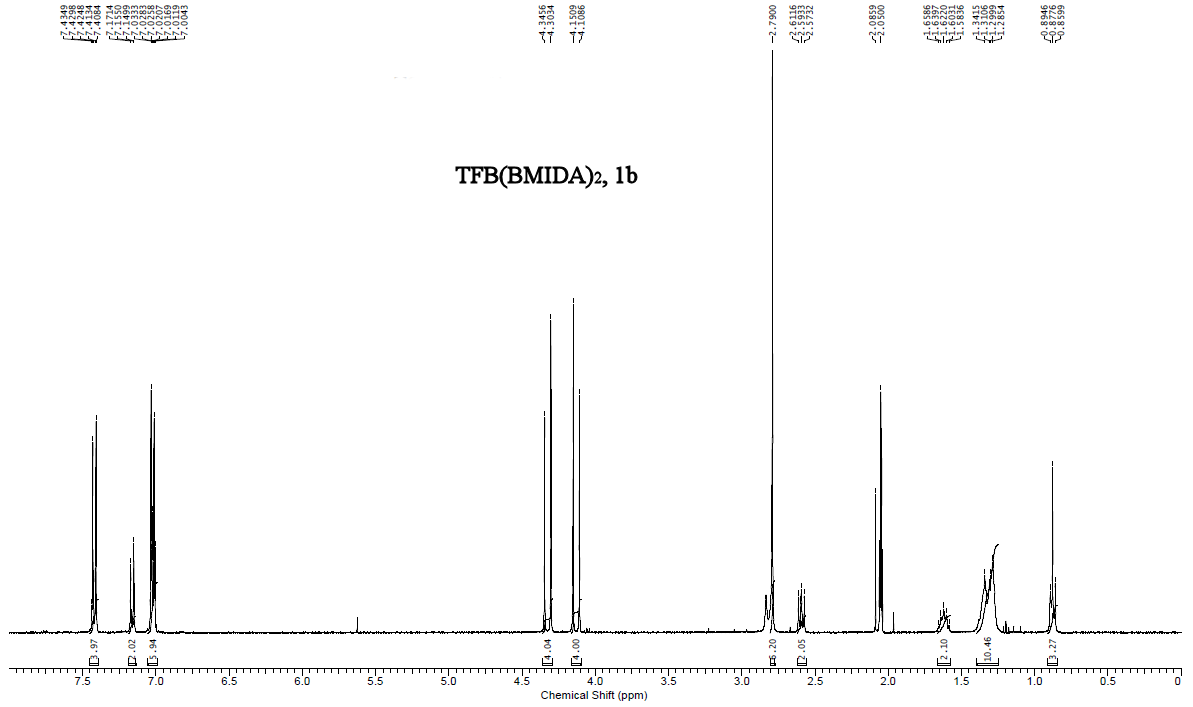
**

**Figure S1.** ^1^H NMR of TFB(BMIDA)_2_ in deuterated acetone.

**Synthesis of PFB(BMIDA)_2_, 2b**

Part 1: To a stirred suspension of anhydrous AlCl_3_ (1.18 g, 8.8 mmol) in anhydrous dichloromethane (10 mL) *N*,*N*-dimethylaniline, termed DMA, (1.12 mL, 8.8 mmol) and BCl_3_ (11 mL of 0.8 m solution in dichloromethane, 8.8 mmol) were successively added at 0–5 °C (ice-water bath, this cooling is sufficient to keep the reaction below 40^o^C) under argon atmosphere (**CAUTION** mildly exothermic reaction cooling required, particularly if preparing on a large scale). The cooling bath was then removed and the reaction mixture was stirred at ambient temperature until complete homogenisation (~1 h). Then, 1,4-bis[N-4-(oct-1-yl)phenyl-N-phenylamino]benzene (**PFB**) (2.55 g, 4 mmol) was added all at once and the reaction mixture was stirred at ambient temperature for another 2-24 h to accomplish formation of a bis-dichloroborane. Then, it was trans-esterified into MIDA-boronate (**PFB(BMIDA)_2_, 2b**) as follows.

Part 2: The mixture from Part 1 was diluted with anhydrous dichloromethane (20 mL), chilled to 0–5 °C and *N*,*N'*-dimethyl-1,3-imidazolin-2-one, DMI (1.89 mL, 17.6 mmol) (**CAUTION** mildly exothermic reaction cooling required, particularly if preparing on a large scale) and bis-TMS-MIDA (2.56 g, 80 mmol) were successively added by cannula with vigorous stirring. The cooling was removed and the reaction mixture was allowed to warm up to room temperature. After 24 h, the supernatant solution was filtered in an inert atmosphere and the residue was washed with dry dichloromethane (3x20 mL). The crude product was dried in vacuo to remove residual dichloromethane. Then, it was dissolved in hot acetone and passed through thin plug of celite. The filtrate was concentrated and left in a dark place for crystallisation. Filtration, washing with cold acetone and drying *in vacuo* (0.02 mbar, 24 h) afforded pure product as off-white free-flowing powder.

According to Part 1, 1,4-bis[N-4-(oct-1-yl)phenyl-N-phenylamino]benzene (**PFB**) (2.55 g, 4 mmol) afforded 2.2 g (58%) of pure product as a colourless free-flowing powder (**PFB(BMIDA)_2_, 2b**)

**^1^H NMR** (400 MHz, acetone-*d*_6_, δ, ppm): 7.42–7.38 (m, 4 H), 7.18–7.14 (m, 4 H), 7.06–6.98 (m, 8 H), 7.00 (s, 4 H), 4.32 (d, ^2^*J*_(H,H)_ = 17.0 Hz, 4 H), 4.12 (d, ^2^*J*_(H,H)_ = 17.0 Hz, 4 H), 2.77 (s, 6 H, C*H_3_*N), 2.58 (dd, ^3^*J*_(H,H)_ = 7.8, 7.7 Hz, 4 H, -C*H_2_*-Ar), 1.65–1.57 (m, 4 H, C*H_2_*CH_2_-Ar), 1.39–1.23 (m, 20 H), 0.87 (t, ^3^*J*_(H,H)_ = 6.9 Hz, 6 H, CH_3_)

**^13^C NMR** (100.6 MHz, acetone-*d*_6_, δ, ppm): *δ* 169.3 (4 C_quat_), 149.8 (2 C_quat_), 146.1 (2 C_quat_), 143.9 (2 C_quat_), 138.8 (2 C_quat_), 134.3 (4 CH), 130.2 (4 CH), 126.3 (4 CH-middle), 125.5 (4 CH), 122.5 (4 CH), 62.6 (4 CH_2_), 48.2 (2 CH_3_N), 35.9 (CH_2_-Ar), 32.6 (CH_2_), 32.4 (CH_2_), 30.2 (CH_2_), 30.1 (CH_2_), 30.0 (CH_2_), 23.3 (CH_2_), 14.4 (CH_3_)

**^11^B NMR** (128.4 MHz, acetone-*d*_6_, δ, ppm): *δ* 11.7 (s)

**HRMS (ESI)**: calcd. for C_56_H_67_B_2_N_4_O_8_ [M – H^+^] 945.7752, found . 945.7

**
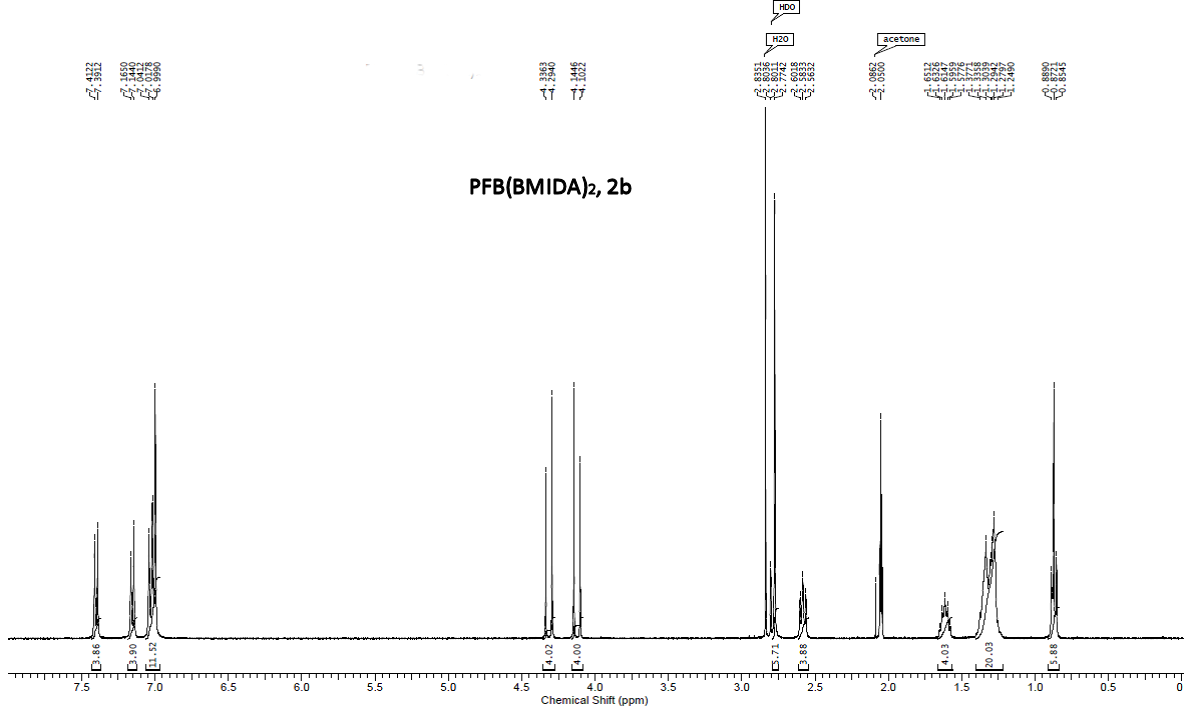
**

**Figure S2.** ^1^H NMR of PFB(BMIDA)_2_ in deuterated acetone.

**^1^H NMR analysis of copolymers, PTFB, PPFB, PF8TFB and PF8PFB**

Poly (4-octylphenyldiphenylamine-alt-4-sec-butylphenyldiphenylamine) (**PTFB**) (400 mHz, CDCl_3_, δ, ppm): 7.58-7.37 (d, 8H, aromatic H), 7.22-6.99 (m, 16 H, aromatic H), 2.68-2.49 (br, 3 H, CH_2_, CH), 1.69-1.51 (br, 4 H, 2 × CH_2_), 1.42-1.17 (m, 13 H, CH_3_, (CH_2_)_5_)), 0.96-0.77 (m, 6H, 2 × CH_3_) (assignments detailed in Figure S3).

Poly (N, N’-bis(4-octylphenyl)-N, N’-diphenylphenylenediamine ) (**PPFB**) (400 mHz, CDCl_3_, δ, ppm): 7.52-7.35 (d, 4H, aromatic H), 7.20-6.89 (m, 16H, aromatic H), 2.57 (t, 4H, 2 × CH_2_), 1.70-1.51 (br, 4H, 2 × CH_2_), 1.42-1.17 (br, 20H, 2 × (CH_2_)_5_), 0.88 (t, 6H, 2 × CH_3_) (assignments detailed in Figure S4).

Poly (9, 9-dioctylfluorene-alt-4-octylphenyldiphenylamine) (**PF8TFB**) (400 mHz, CDCl_3_, δ, ppm): 7.79-7.71 (d, 2H, aromatic H), 7.64-7.51 (m, 8H, aromatic H), 7.26-7.19 (d, 4H, aromatic H), 7.18-7.10 (m, 4H, aromatic H), 2.66-2.54 (m, 2H, CH_2_), 2.10-1.95 (br, 4H, 2 × CH_2_), 1.80-1.45 (br, 2 H, TFB CH_2_), 1.42-1.23 (m, 10 H, TFB (CH_2_)_5_), 1.23-0.99 (m, 20H, F8 2 × (CH_2_)_5_), 0.89 (t, 3H, TFB CH_3_), 0.80 (t, 6H, F8 2 × CH_3_), 0.76-0.64 (br, 4H, F8 2 × CH_2_) (assignments detailed in Figure S5).

Poly (9, 9-dioctylfluorene-alt-N, N’-bis(4-octylphenyl)-N, N’-diphenylphenylenediamine) (**PF8PFB**) (400 mHz, CDCl_3_, δ, ppm): 7.79-7.67 (d, 2H, aromatic H), 7.62-7.47 (br, 4H, aromatic H), 7.24-6.90 (m, 20H, aromatic H), 2.68-2.49 (br, 4H, PFB 2 × CH_2_), 2.10-1.92 (br, 4H, F8 2 × CH_2_), 1.43-1.22 (m, 24H, PFB 2 × (CH_2_)_5_, 2 × CH_2_), 1.21-0.99 (m, 20H, F8 2 × (CH_2_)_5_), 0.89 (t, 6H, PFB CH_3_), 0.79 (t, 6H, F8 2 × CH_3_), 0.75-0.61 (br, 4H, F8 2 × CH_2_) (assignments detailed in Figure S6).

**
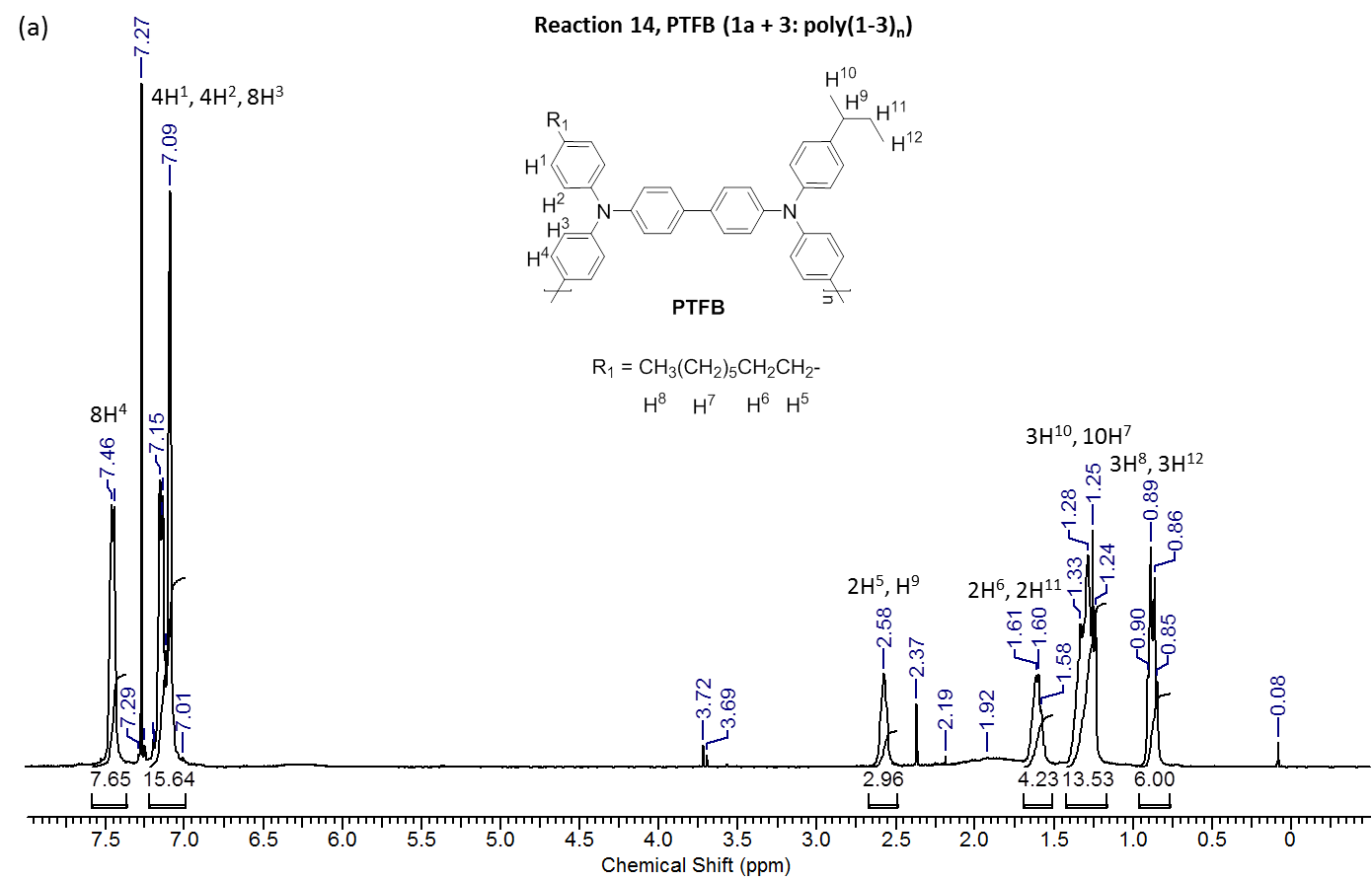
**

**
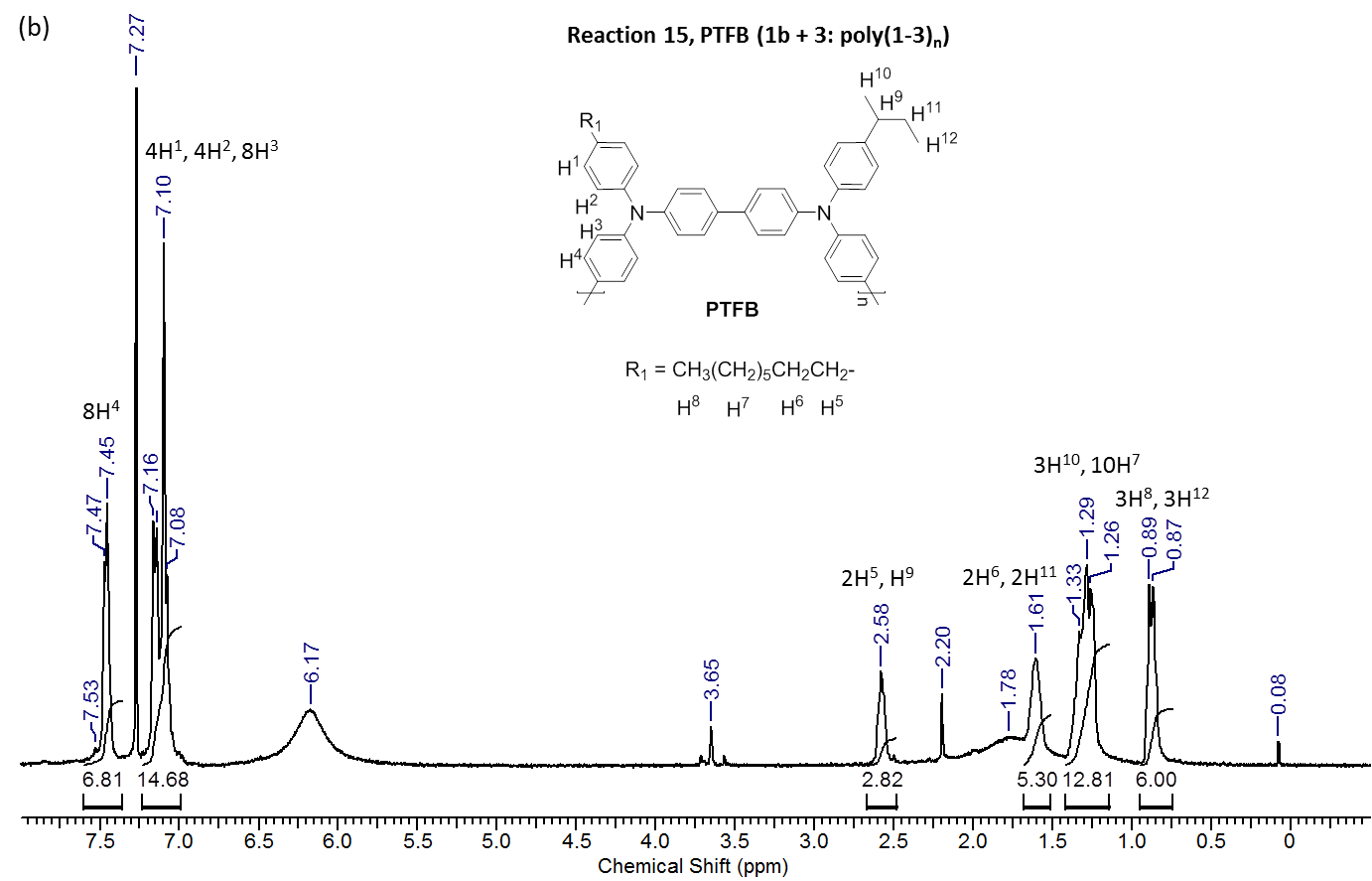
**

**Figure S3.** ^1^H NMR spectra of PTFB produced in (a) Reaction 14 (**1a** + **3**: poly(**1-3**)_n_) and (b) Reaction 15 (**1b** + **3**: poly(**1-3**)_n_) in CDCl_3_

**
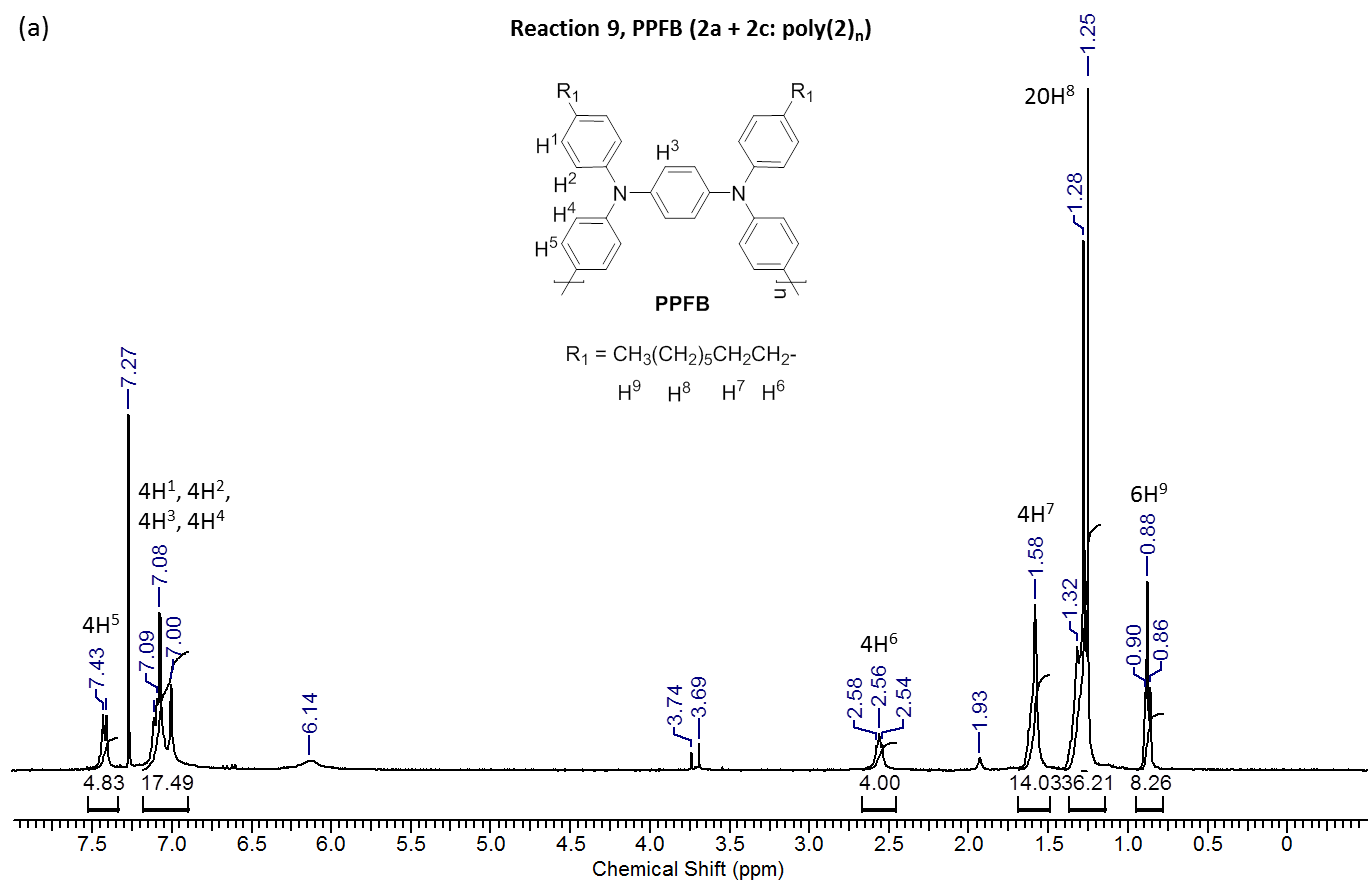
**

**
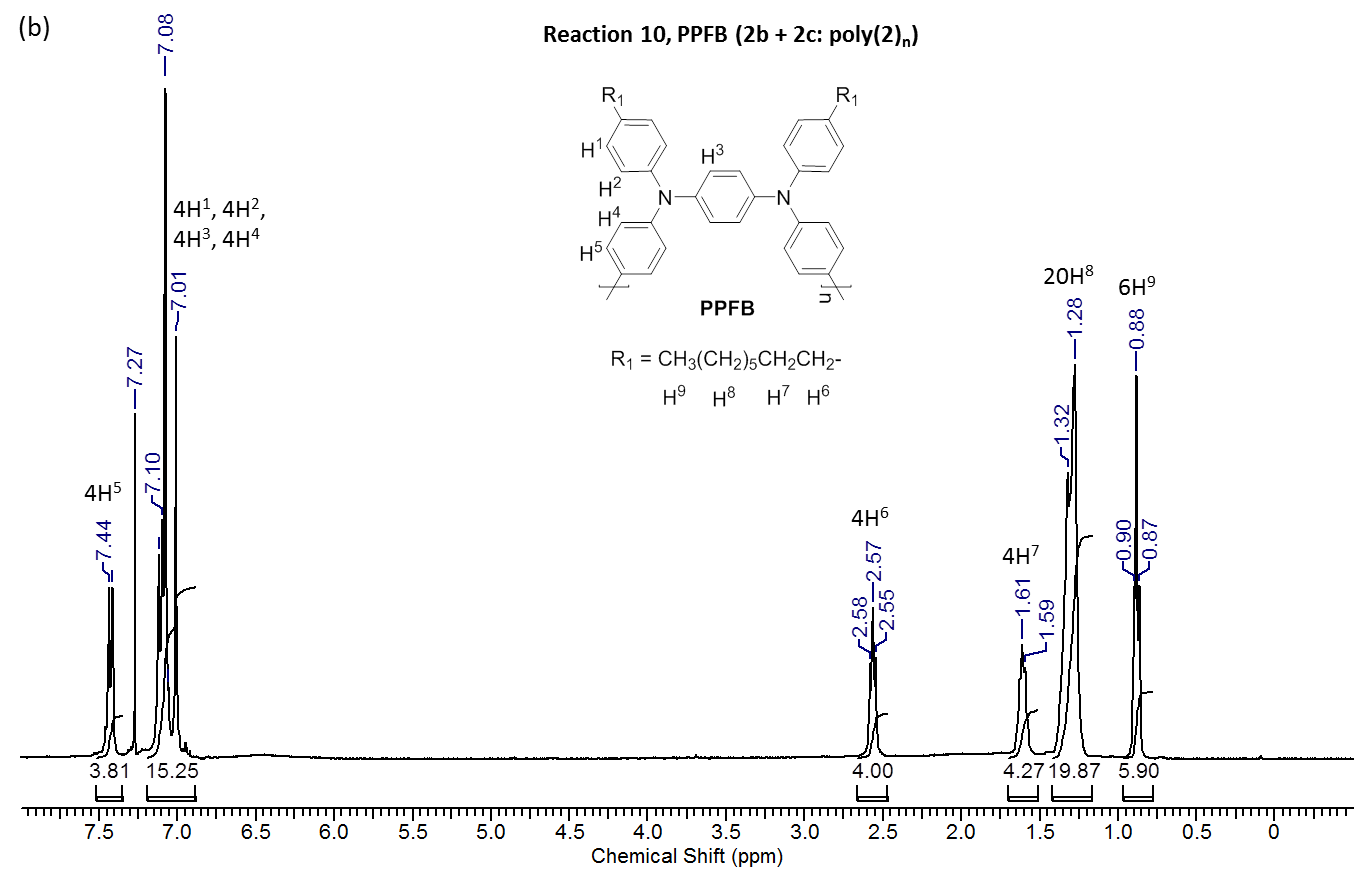
**

**Figure S4.** ^1^H NMR spectra of PPFB produced in (a) Reaction 9 (**2a** + **2c**: poly(**2**)_n_) and (b) Reaction 10 (**2b** + **2c**: poly(**2**)_n_) in CDCl_3_

**
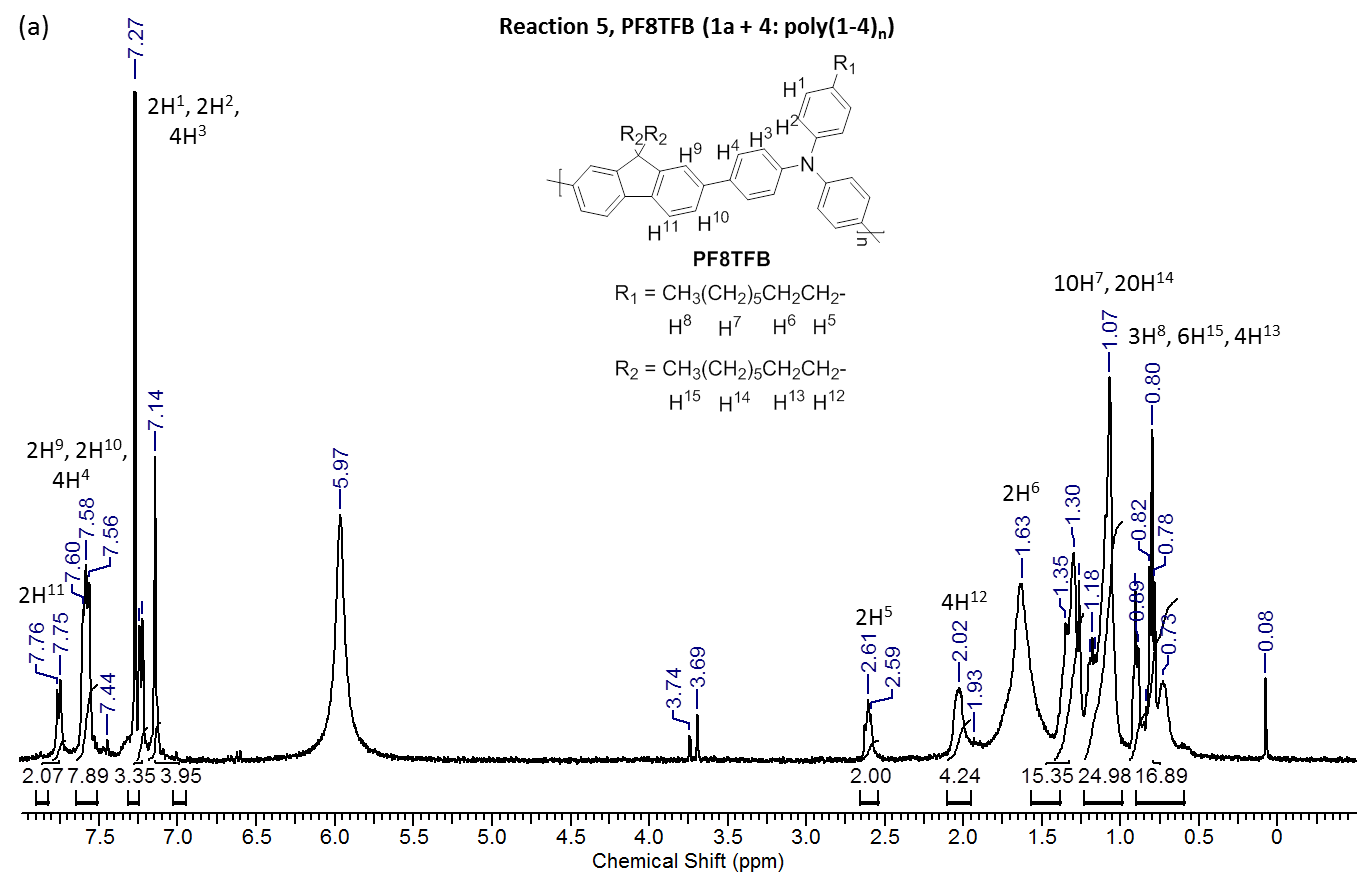
**

**
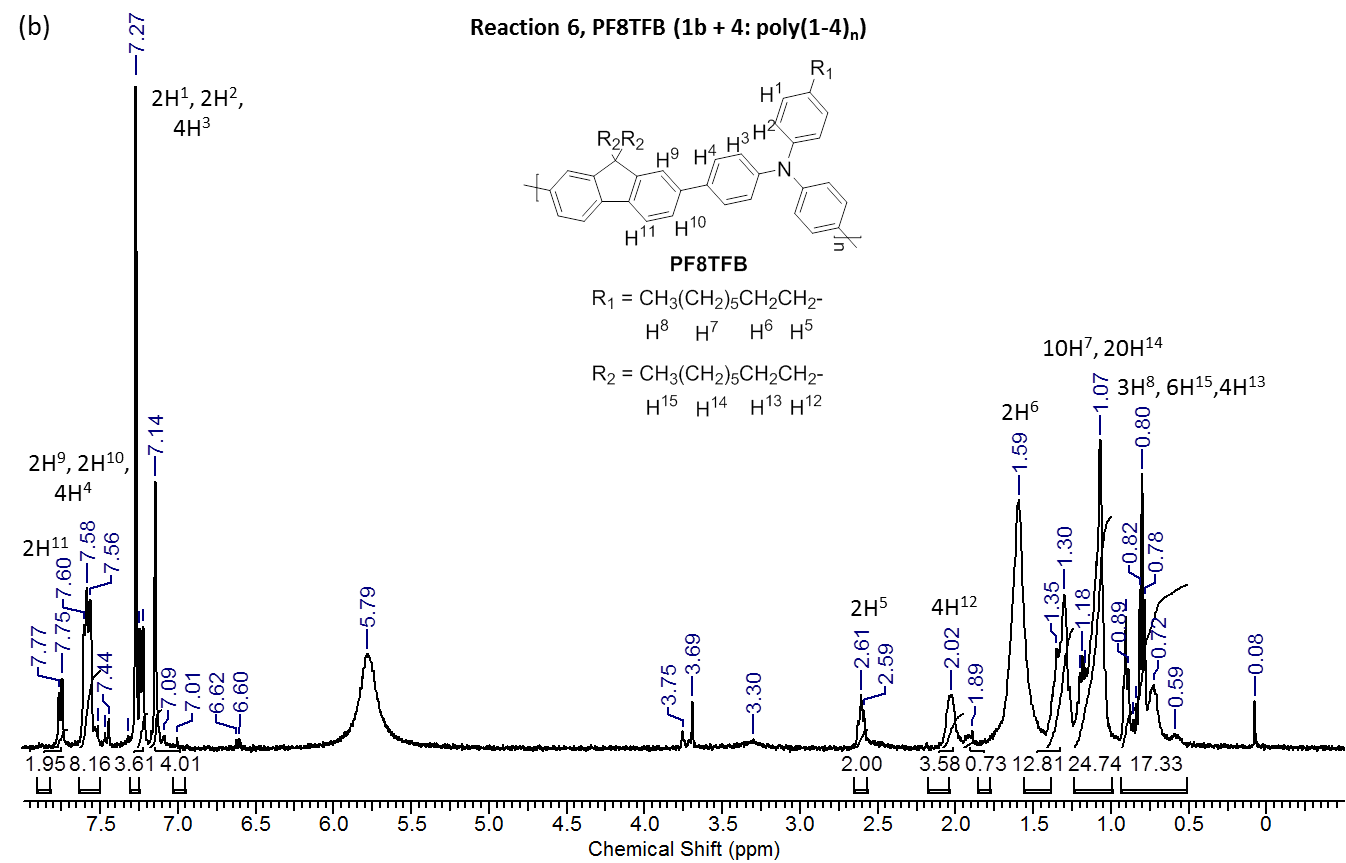
**

**Figure S5.** ^1^H NMR spectra of PF8TFB produced in (a) Reaction 5 (**1a** + **4**: poly(**1-4**)_n_) and (b) Reaction 6 (**1b** + **4**: poly(**1-4**)_n_) in CDCl_3_

**
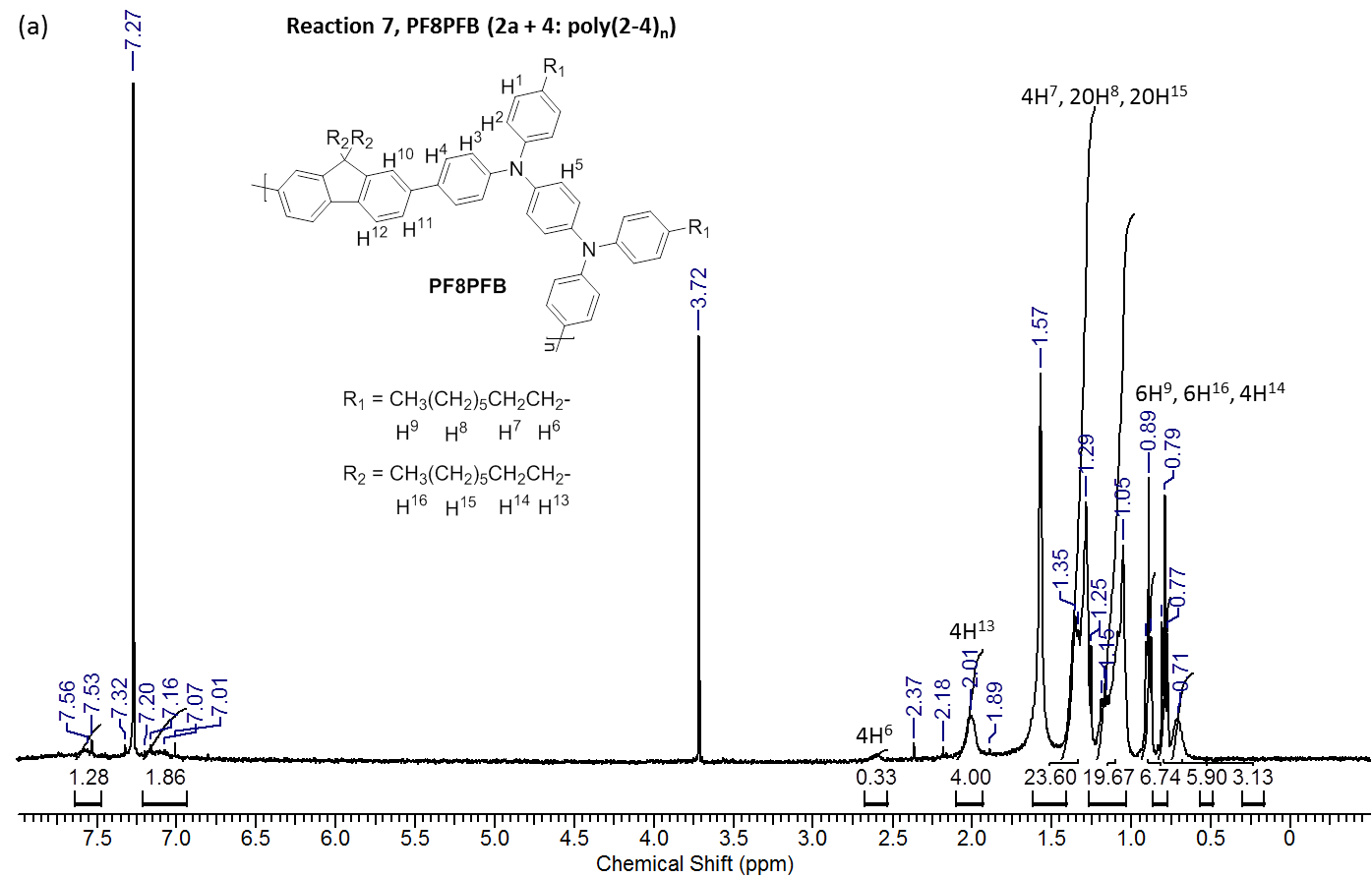
**

**
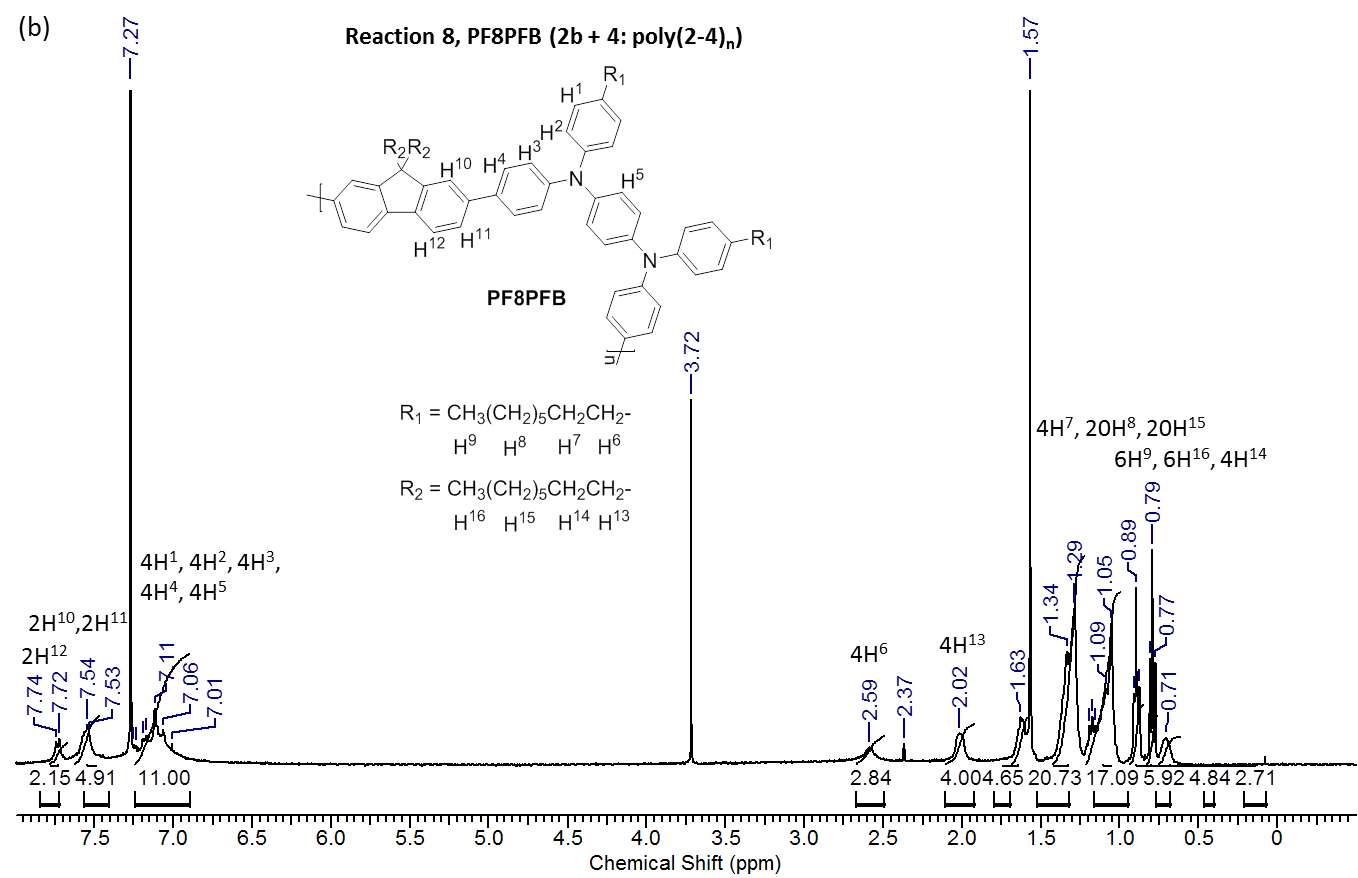
**

**Figure S6.** ^1^H NMR spectra of PF8PFB produced in (a) Reaction 7 (**2a** + **4**: poly(**2-4**)_n_) and (b) Reaction 8 (**2b** + **4**: poly(**2-4**)_n_) in CDCl_3_

**Further GPC analysis of copolymers**


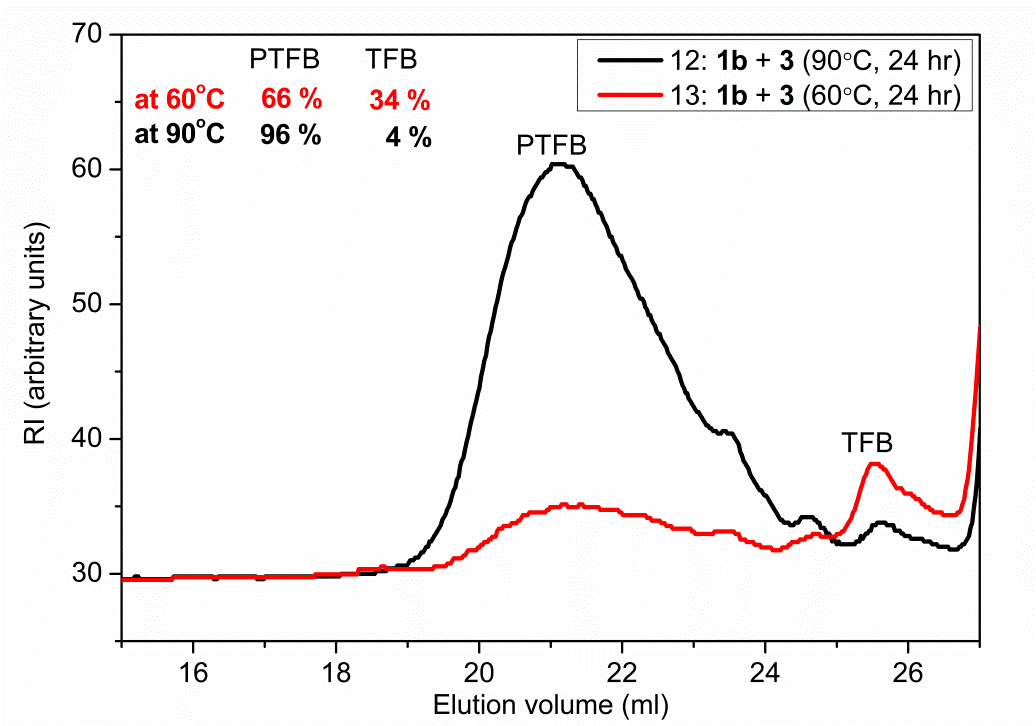


**Figure S7.** GPC chromatograms of polymers obtained in cross-coupling reactions of bis MIDA boronate ester TFB monomer (**1b**) with dibromo TFB monomer (**3**) after 24 hours at 60 and 90 °C respectively (reactions 13 & 12). The conversion percentages are calculated from the respective integral areas of peaks associated with the monomer (TFB) and the polymer (PTFB).


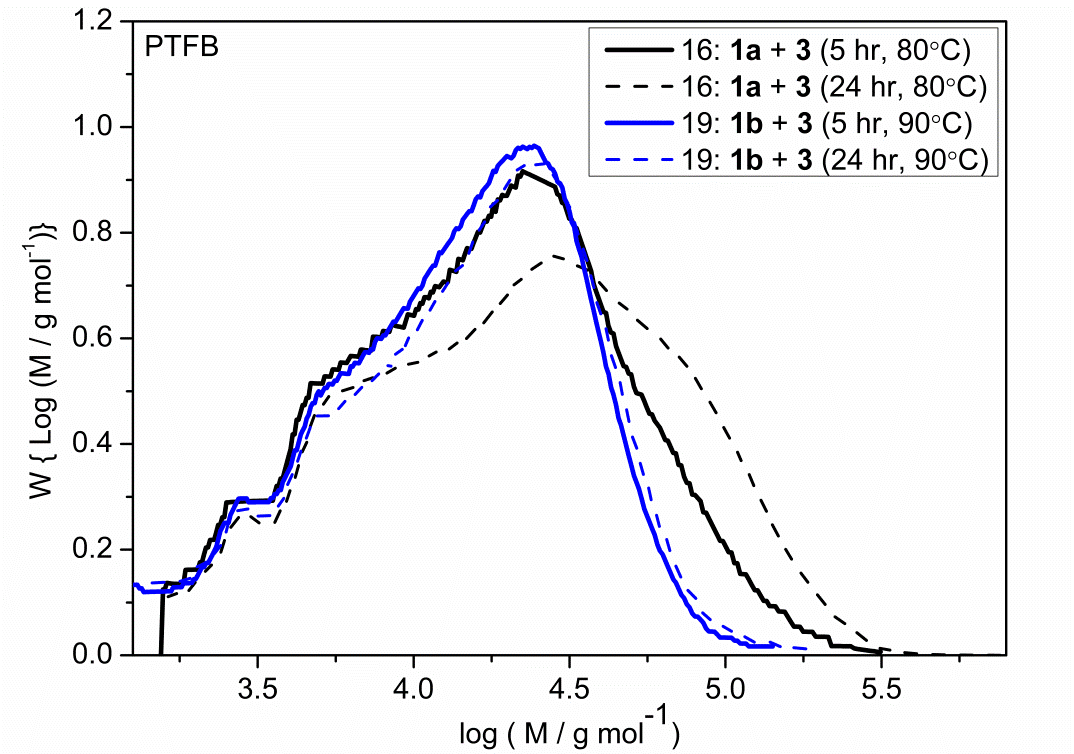


**Figure S8.** GPC molar mass distributions of PTFB polymers obtained from cross-coupling reactions of bis pinacol ester or bis BMIDA functionalized TFB monomer (**1a** or **1b**) with dibromo TFB monomer (**3**) in toluene / dioxane / water, 1: 1: 1 (ml) solvent mixtures at 80 or 90 °C respectively, after different time intervals (reactions 16 & 19)

**MALDI analysis of copolymers**

**Table S1.** Expected (Exp.) and Actual (Act.) MALDI peak assignments of PPFB (Figure 2).

| 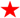 | [H−(**2**)_n_−H]^+^ | **n** | 3 | 4 | 5 | 6 | 7 | 8 | 9 | 10 | 11 | 12 | 13 |
| --- | --- | --- | --- | --- | --- | --- | --- | --- | --- | --- | --- | --- | --- |
|  |  | **Exp.** | 1908 | 2543 | 3178 | 3813 | 4448 | 5083 | 5718 | 6353 | 6988 | 7623 | 8258 |
|  |  | **Act.** | 1910 | 2543 | 3180 | 3813 | 4449 | 5083 | 5718 |  | 6986 |  | 8255 |
| 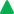 | [H−(**2**)_n_−Br]^+^ | **n** | 3 | 4 | 5 | 6 | 7 | 8 | 9 | 10 | 11 | 12 |  |
|  |  | **Exp.** | 1987 | 2622 | 3257 | 3892 | 4527 | 5162 | 5797 | 6432 | 7067 | 7702 |  |
|  |  | **Act.** | 1985 | 2624 | 3257 | 3893 | 4528 |  |  | 6432 |  |  |  |

**Table S2.** Expected (Exp.) and Actual (Act.) MALDI peak assignments of PTFB (Figure 4(a)).

| 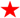 | [H−(**1**−**3**)_n_−H]^+^ | **n** | 3 | 4 | 5 | 6 | 7 | 8 |
| --- | --- | --- | --- | --- | --- | --- | --- | --- |
|  |  | **Exp.** | 1968 | 2623 | 3278 | 3933 | 4588 | 5243 |
|  |  | **Act.** | 1968 | 2624 | 3279 | 3935 | 4589 | 5244 |
| 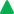 | [H−(**1**−**3**)_n_−**1**−H]^+^ | **n** | 3 | 4 | 5 | 6 | 7 |  |
|  |  | **Exp.** | 2324 | 2979 | 3634 | 4289 | 4944 |  |
|  |  | **Act.** | 2324 | 2980 | 3635 | 4290 | 4945 |  |
| 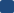 | [H−**3**−(**1**−**3**)_n_−H]^+^ | **n** | 3 | 4 | 5 |  |  |  |
|  |  | **Exp.** | 2267 | 2922 | 3577 |  |  |  |
|  |  | **Act.** | 2269 | 2925 | 3581 |  |  |  |
| 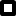 | [H−(**1**)_2_−(**1**−**3**)_n_−H]^+^ | **n** | 2 | 3 | 4 | 5 | 6 |  |
|  |  | **Exp.** | 2025 | 2680 | 3335 | 3990 | 4645 |  |
|  |  | **Act.** | 2026 | 2681 | 3336 | 3991 | 4647 |  |

**Table S3.** Expected (Exp.) and Actual (Act.) MALDI peak assignments of PFTB (Figure 4(b)).

|  | [H−(**1**)_3_−(**1**−**3**)_n_−H]^+^ | **n** | 4 | 5 | 6 |
| --- | --- | --- | --- | --- | --- |
|  |  | **Exp.** | 3691 | 4346 | 5001 |
|  |  | **Act.** | 3692 | 4347 | 5002 |
| 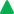 | [H−(**1**−**3**)_n_−**1**−H]^+^ | **n** | 5 | 6 | 7 |
|  |  | **Exp.** | 3634 | 4289 | 4944 |
|  |  | **Act.** | 3635 | 4291 | 4945 |
|  | [Br−**3**−(**1**)_4_−(**1**−**3**)_n_−Br]^+^ | **n** | 3 | 4 | 5 |
|  |  | **Exp.** | 3849 | 4504 | 5159 |
|  |  | **Act.** | 3849 | 4504 | 5157 |
| 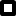 | [H−(**1**)_2_−(**1**−**3**)_n_−H]^+^ | **n** | 5 | 6 | 7 |
|  |  | **Exp.** | 3990 | 4645 | 5300 |
|  |  | **Act.** | 3992 | 4646 | 5300 |
| **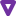** | [H−(**1**)_4_−(**1**−**3**)_n_−H]^+^ | **n** | 4 | 5 | 6 |
|  |  | **Exp.** | 4047 | 4702 | 5357 |
|  |  | **Act.** | 4048 | 4702 | 5357 |
| 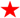 | [H−(**1**−**3**)_n_−H]^+^ | **n** | 6 | 7 | 8 |
|  |  | **Exp.** | 3933 | 4588 | 5243 |
|  |  | **Act.** | 3935 | 4589 | 5244 |
|  | [H−(**3**)_2_−(**1**−**3**)_n_−H]^+^ | **n** | 5 | 6 |  |
|  |  | **Exp.** | 3876 | 4531 |  |
|  |  | **Act.** | 3872 | 4529 |  |
| 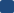 | [H−**3**−(**1**−**3**)_n_−H]^+^ | **n** | 5 | 6 |  |
|  |  | **Exp.** | 3577 | 4232 |  |
|  |  | **Act.** | 3577 | 4232 |  |

**Scale-up PF8PFB reaction (larger scale version of Reaction 23)**

The conditions used for the larger scale Suzuki-Miyaura cross-coupling reaction of equimolar amounts of **2b** and **4** (0.12 mmol) at 90 °C for 24 hr are outlined below.

**Experimental details**

Equimolar amounts of monomers, **2b** (1.136 g, 1.2 mmol) and **4** (0.658 g, 1.2 mmol) were placed into a 100 ml three necked round-bottom flask. Amounts of toluene (10.00 ml, 8.65 g) and dioxane (12.00 ml, 12.41 g) were next washed into the flask. A 20 ml stock solution of potassium phosphate tribasic (K_3_PO_4_) was prepared (consisting of 0.1592 g, 7.5 mmol K_3_PO_4_ per 1 ml water). An amount of this base solution (13.91 g) was transferred into the flask. The tube contents were then stirred with a magnetic stirring bar whilst being thoroughly degassed by bubbling nitrogen gas through the solution for 30 minutes.

A solution of the palladium (II) acetate [Pd(OAc)_2_] / 2-dicyclohexylphosphino-2’,6’-dimethoxybiphenyl (SPhos) catalyst system in toluene was prepared in a small round-bottomed flask. The solution was composed of: Pd(OAc)_2_ (13.44 mg, 0.06 mmol), SPhos (49.32 mg, 0.12 mmol) and toluene (2.0 ml, 1.73 g). The catalyst solution in the flask was stirred with a magnetic flea for about 20 minutes whilst being thoroughly degassed by three repeated cycles of evacuation followed by replenishment with nitrogen gas.

The previously degassed reaction flask and contents were heated to 90 °C. A glass syringe was used to inject the entire catalyst solution into the heated flask contents whilst maintaining a nitrogen environment. The flask contents were stirred under a nitrogen environment at 90 °C for a period of 24 hours. A sample of the reaction mixture (0.5 ml) was removed after 5 hours under a nitrogen atmosphere using a glass syringe and placed into a small amount of toluene (1.0 ml). The sample solution was allowed to cool, before being added dropwise to a stirred excess amount of chilled methanol (5 ml) to precipitate the polymer. The polymer formed a fine dispersal in the stirred methanol. A pipette was used to transfer samples of this dispersal into a pair of vials suitable for use in a centrifuge. The vial samples were placed in a centrifuge at 14,000 rpm for 10 minutes. The supernatant layer was removed from above the separated polymer in the vials. The polymer samples in the vials were then dried and re-dissolved in THF for GPC analysis.

After 24 hours, upon completion of the reaction, the remaining flask contents were added to a small amount of toluene (10.0 ml). The diluted reaction solution was then allowed to cool, before being added dropwise to a stirred excess amount of chilled methanol (250 ml) to precipitate the polymer. Small samples of the dispersal obtained were then removed and treated in the same way to prepare polymer samples for GPC analysis.

The precipitated polymer solution was filtered through a sintered glass funnel under vacuum. The collected polymer was re-dissolved in toluene (20 ml) and the resultant solution then washed repeatedly with 0.05M sodium diethyldithiocarbamate solution to remove any remnants of the palladium catalyst. The solution was then added dropwise to a stirred excess amount of chilled methanol (250 ml) to precipitate the polymer. The precipitated polymer solution was filtered through a sintered glass funnel under vacuum. The polymer sample was then dried further under vacuum at room temperature until constant mass was obtained.

The yield of polymer obtained from the larger scale cross-coupling reaction of **2b** with **4**, after the washing and re-precipitation steps, was 0.98 g (80 %). The molar masses of the polymers obtained from both reactions completed with these monomers under the same conditions are compared in **Table S4**. The molar mass distributions of the two polymers obtained are overlaid in **Figure S9**. The larger scale cross-coupling reaction produced polymer of marginally higher molar mass (M_n_ = 23k, M_p_ = 81k) in a narrower distribution (Ð = 2.8).

**Table S4.** GPC analysis of the molar masses of polymers obtained from Suzuki cross-coupling reactions of **2b** and **4** in toluene / dioxane / water (1: 1: 1 ml) solvent mixtures in presence of Pd(OAc)_2_ / SPhos (2.5 / 5.0 mol %) catalyst system at 90 °C after 24 hr.

| Entry | **2b**  / mmol | **4**  / mmol | M_p_  / kg mol^-1^ | M_w_  / kg mol^-1^ | M_n_  / kg mol^-1^ | Ð |
| --- | --- | --- | --- | --- | --- | --- |
| 23 | 0.1 | 0.1 | 77.0 | 59.4 | 20.0 | 3.0 |
| 23 (scale-up) | 1.2 | 1.2 | 81.4 | 65.9 | 23.4 | 2.8 |

**Figure S9.** Molar mass distributions of PF8PFB polymers obtained in cross-coupling reactions of **2b** with **4** at 90 °C after 24 hours (reactions 23 & 23 scale-up).

**References**

1. Bagutski, V.; Del Grosso, A.; Carrillo, J. A.; Cade, I. A.; Helm, M. D.; Lawson, J. R.; Singleton, P. J.; Solomon, S. A.; Marcelli, T.; Ingleson, M. J. J. Am. Chem. Soc. 2013, 135, 474-487.
